# Disentangling Dose and Radiation Quality Effects on Late DNA Damage Response in Human Mesenchymal Stromal Cells

**DOI:** 10.3390/ijms27219423

**Published:** 2026-10-22

**Authors:** Sofia A. Korneva, Alexander P. Chernyaev, Oleg V. Belov, Anna Chigasova, Andrey А. Osipov, Maxim Ignatov, Elizaveta Yashkina, Vyacheslav Saburov, Natalia Vorobyeva, Nikolay Zyuzikov, Andreyan N. Osipov

**Affiliations:** 1 Faculty of Physics, Lomonosov Moscow State University, 119991 Moscow, Russia; sophiakorneva@gmail.com (S.A.K.); a.p.chernyaev@yandex.ru (A.P.C.); 2 State Research Center—Burnasyan Federal Medical Biophysical Center of Federal Medical Biological Agency (SRC—FMBC), 123098 Moscow, Russia; annagrekhova1@gmail.com (A.C.); mantroz@yandex.ru (M.I.); yashkinaliz@gmail.com (E.Y.); nuv.rad@mail.ru (N.V.); 3 Skobeltsyn Institute of Nuclear Physics, Lomonosov Moscow State University, 119991 Moscow, Russia; 4 Veksler and Baldin Laboratory of High Energy Physics, Joint Institute for Nuclear Research, 141980 Dubna, Russia; oleg.belov@jinr.int; 5 Institute of Biomedical Problems, Russian Academy of Sciences, 123007 Moscow, Russia; 6 N.N. Semenov Federal Research Center for Chemical Physics, Russian Academy of Sciences, 119991 Moscow, Russia; a-2-osipov@yandex.ru; 7 Emanuel Institute for Biochemical Physics, Russian Academy of Sciences, 119334 Moscow, Russia; 8 A. Tsyb Medical Radiological Research Center, Branch of the National Medical Research Radiological Center of the Ministry of Health of the Russian Federation, 249031 Obninsk, Russia; vosaburov@gmail.com; 9 Department of Physics, Faculty of Science and Technology, The University of the West Indies, St. Augustine 999183, Trinidad and Tobago; nikolay.zyuzikov@sta.uwi.edu; 10 CANDLE Synchrotron Research Institute, 31 Acharyan, Yerevan 0040, Armenia

**Keywords:** γH2AX, pATM, RAD51, DNA double-strand breaks, DNA repair kinetics, radiation quality, linear energy transfer (LET), neutrons, protons, kinetic modeling

## Abstract

Although ionizing radiation quality shapes the spatial structure and complexity of primary DNA lesions, its contribution relative to absorbed dose in the late DNA damage response (DDR) remains poorly quantified. We analyzed γH2AX and pATM foci kinetics in human mesenchymal stromal/stem cells exposed to ^60^Co γ-rays, 150 MeV protons, and 14.1 MeV neutrons at doses of 0.25, 0.5, 1.0, and 2.0 Gy, delivered at dose rates of 0.5 Gy/min for γ-rays, 1 Gy/min for protons, and 46 mGy/min for neutrons, respectively. An effective kinetic model was used to describe the time course of the DDR and incorporated an ultraslow component as a model-based index of long-lasting repair burden. Radiation types differed less in early signal amplitude than in decay rate and late persistence. At every investigated dose, the normalized ultraslow fraction followed the order γ-rays < protons < neutrons. This ordering was preserved in per-dose fits, while parametric bootstrapping supported the γ–neutron separation, although the absolute values of the internal fractions were only partially identifiable. RAD51, analyzed independently from model fitting, showed the same qualitative ordering at late time points, supporting the interpretation that radiation quality is more associated with persistent DDR signaling than absorbed dose within the investigated range. These findings suggest that absorbed dose alone may not fully characterize the late cellular response to different radiation qualities and highlight the importance of radiation quality in comparative radiobiology, radiation protection, and the interpretation of normal-tissue responses to therapeutic irradiation.

## 1. Introduction

Ionizing radiation induces a broad spectrum of DNA lesions, among which double-strand breaks (DSBs) are considered particularly hazardous because simultaneous disruption of both DNA strands can compromise DNA continuity and increase the risk of erroneous end joining, chromosome rearrangements, and loss of genetic information. Incomplete or inaccurate DSB repair can therefore promote genomic instability and alter cell fate. To counter such damage, cells activate the DNA damage response (DDR), a coordinated signaling network that integrates lesion recognition, signal transduction, cell-cycle control, and DNA repair [1,2]. The intensity and duration of the DDR depend not only on the number of primary lesions but also on their structural complexity, cellular context, and accessibility to the repair machinery. For example, DSBs arising in densely packed chromatin may require chromatin remodeling and spatial reorganization before repair can be completed [3,4].

Phosphorylated ATM (pATM) and phosphorylated histone H2AX (γH2AX) are widely used indicators of radiation-induced DDR activation [5,6,7]. ATM is a central regulator of the response to DSBs and coordinates damage signaling, checkpoint activation, and repair [8], whereas H2AX phosphorylation around DNA damage sites generates γH2AX domains that facilitate the accumulation and retention of DDR and repair proteins [9]. Because pATM and γH2AX can be visualized and quantified as nuclear foci by immunofluorescence microscopy, their kinetics are commonly used to characterize radiation-induced DDR [10,11,12]. However, neither marker provides a direct one-to-one measure of the number of unrepaired DSBs. Their temporal dynamics reflect the combined effects of lesion recognition, ATM-dependent signaling, spreading and resolution of γH2AX-associated chromatin domains, recruitment and release of repair factors, and progression of lesion processing and repair [13,14,15].

In radiobiology, radiation quality refers to the physical characteristics that determine the spatial pattern of energy deposition in matter, including linear energy transfer (LET), ionization density, and track structure. At the same absorbed dose, different radiation types can therefore generate distinct spectra of primary DNA lesions because the local density and spatial organization of energy deposition differ. In the present study, “radiation quality” is used in this sense, referring not to the total energy transferred but to how locally and densely that energy is deposited within the cellular target. These differences influence both the structural complexity of primary lesions and the subsequent kinetics of the DNA damage response.

Densely ionizing radiation is more likely to generate complex or clustered DNA damage, including DSBs accompanied by nearby single-strand breaks, base damage, or chemically modified DNA ends [16,17,18,19,20,21,22]. Such lesions may require additional end processing and coordinated repair steps before resolution, which can contribute to the prolonged persistence of DDR-associated markers [23,24,25,26].

The three radiation modalities were selected to provide a comparative range of radiation qualities within the same cellular and experimental system. ^60^Co γ-rays served as a conventional low-LET photon reference, whereas 150 MeV protons represented a clinically relevant particle-radiation modality. Although 14.1 MeV neutrons are not routinely used in contemporary radiotherapy, they were included as an experimental higher-LET radiation quality. Neutrons of this energy, characteristic of deuterium–tritium neutron generators, produce secondary charged particles with relatively dense ionization patterns in biological matter. The neutron exposure was therefore used as a radiobiological reference condition for investigating the relationship between radiation track structure and the persistence of DDR signaling, rather than as a direct representation of an established clinical neutron-therapy protocol [27,28].

Human mesenchymal stromal/stem cells (hMSCs) are relevant to radiation biology because they contribute to tissue maintenance and regeneration and may therefore affect normal-tissue responses and recovery after irradiation. Previous studies have described hMSCs as relatively radioresistant and have associated this phenotype with efficient DNA damage recognition and repair, robust checkpoint activation, and limited induction of apoptosis [29,30]. Adipose-derived hMSCs were selected here as a normal, non-transformed stromal cell model suitable for examining the persistence and resolution of DDR-associated signaling over a 24-h post-irradiation interval. However, radiosensitivity may vary with tissue source, donor characteristics, passage number, and culture conditions; therefore, the conclusions of the present study are restricted to the investigated adipose-derived hMSC system.

Several kinetic and biophysical models have been developed to describe DNA double-strand break repair and the temporal resolution of radiation-induced damage signals [31,32,33,34,35,36]. Descriptive exponential models capture multiphasic kinetics and estimate fast and slow response timescales, whereas lesion-partition models distinguish rapidly and slowly repairable damage components [32,33,35]. Other frameworks explicitly represent the formation and resolution of radiation-induced foci. For example, Neumaier et al. developed a framework accounting for the induction of DSBs, their conversion into detectable radiation-induced foci, and subsequent focus resolution, with the observed focus yield depending on dose and time after irradiation [14]. More mechanistic ordinary differential equation models represent specific repair pathways, such as non-homologous end joining (NHEJ), through sequential steps involving factor binding, end processing, and ligation [36].

However, these approaches have rarely been applied to the joint analysis of γH2AX and pATM kinetics across different radiation qualities within the same cellular system. This represents an important gap because DDR-marker time courses contain several related but non-equivalent features, including early signal amplitude, decay rate, late residual signal, area under the curve, and the relative contribution of slow components. Consequently, comparisons between radiation qualities require an approach that not only reproduces the experimental curves but also distinguishes early response characteristics from late signal persistence.

In the present study, we used a structured effective kinetic model that does not assign fitted components to specific molecular classes of DNA lesions. Instead, the model represents DDR-marker kinetics through effective damage-associated components coupled to ATM activation and γH2AX signal formation. Its parameters are therefore interpreted as effective kinetic descriptors rather than as literal fractions of distinct lesion types. The central question was whether radiation quality primarily affects the initial magnitude of the DDR or the persistence of the late response in human adipose-derived mesenchymal stromal/stem cells. To address this question, we analyzed γH2AX and pATM foci kinetics after exposure to ^60^Co γ-rays, 150 MeV protons, and 14.1 MeV neutrons at doses of 0.25, 0.5, 1.0, and 2.0 Gy.

As a first step, biexponential fitting was used to characterize the principal timescales of the observed DDR-marker kinetics. We then applied a two-component effective kinetic model linking damage-associated components to ATM activation and γH2AX signal formation. To represent the long-lasting residual response explicitly, the model was extended by introducing an effective ultraslow component. In this study, the term “late-persistent DDR burden” refers to the sustained damage-associated signaling represented by this ultraslow component within the 24-h observation window. Its normalized contribution, *D_u_*_0_/*N*_0_, was used as a comparative model-derived index and not as a direct estimate of the physical fraction of unrepaired DSBs.

Possible biological interpretations of the late-persistent component, including complex or clustered DNA damage and prolonged lesion processing, are considered in the Discussion. RAD51, which was not included in model fitting, was analyzed separately as a complementary biological readout of late repair-associated activity. This allowed us to assess whether an experimentally measured marker outside the fitted γH2AX/pATM system exhibited a radiation-quality-dependent pattern consistent with the model-derived late-persistent DDR index.

Overall, the study was designed to distinguish the contribution of absorbed dose from that of radiation quality to late DDR persistence and to assess the robustness of this distinction across alternative kinetic descriptions. By separating early DDR amplitude from late signal persistence, the proposed framework may contribute to comparative radiobiology, radiation protection, and the interpretation of normal-tissue responses to therapeutic irradiation.

## 2. Results

### 2.1. Experimentally Measured Kinetics of γH2AX and pATM Foci Depend on Radiation Quality

**Figure 1:**
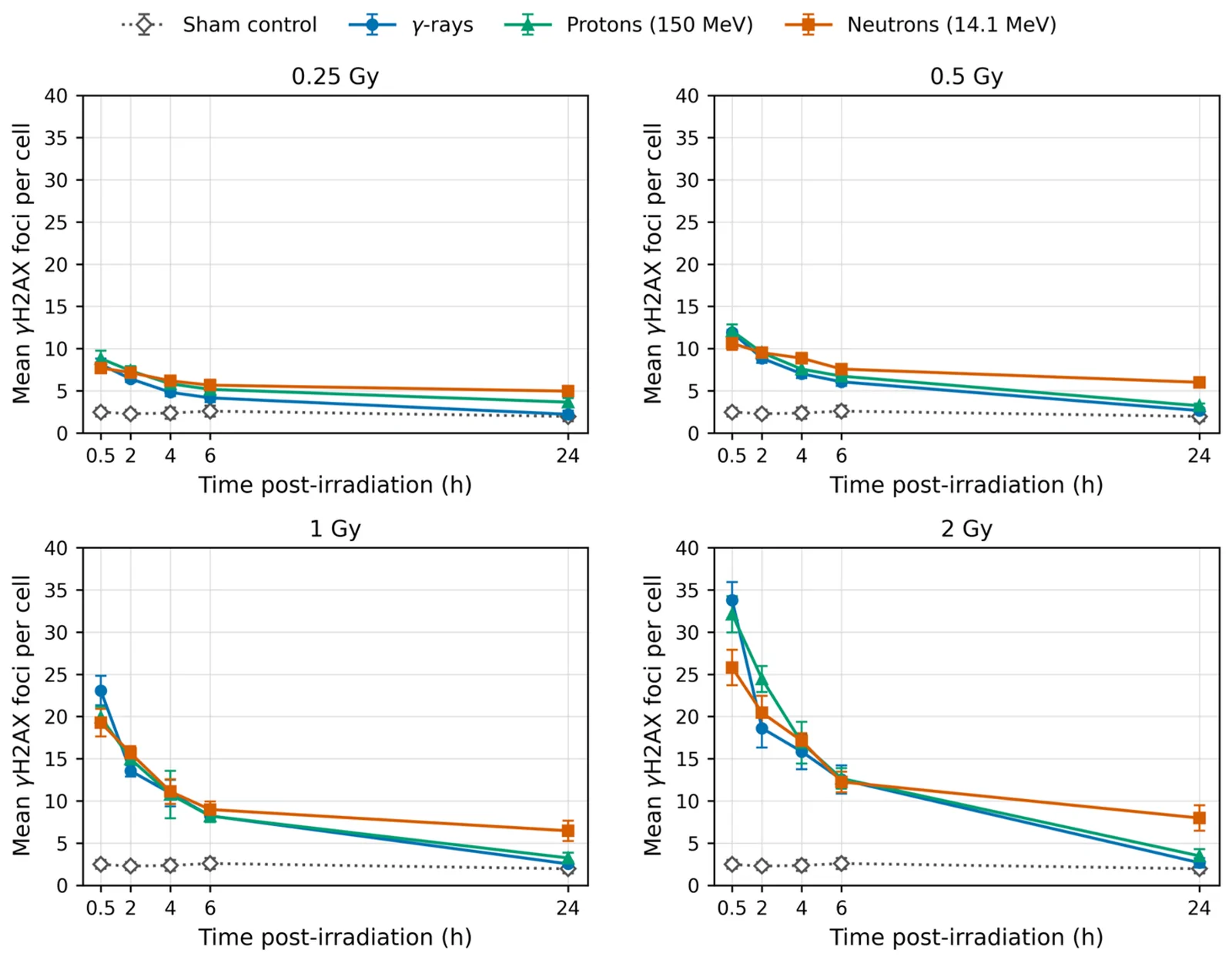
Experimentally measured kinetics of γH2AX foci in hMSCs after irradiation with γ-rays, 150 MeV protons, and 14.1 MeV neutrons at doses of 0.25, 0.5, 1.0, and 2.0 Gy. A common time-matched sham-control series is shown in each panel because all irradiation modalities were examined within the same independent experimental runs. Points represent means from three independent experiments (*n* = 3), and error bars represent standard errors calculated across the independent experimental means. Lines connect experimental means and are shown only to guide the eye.

**Figure 2:**
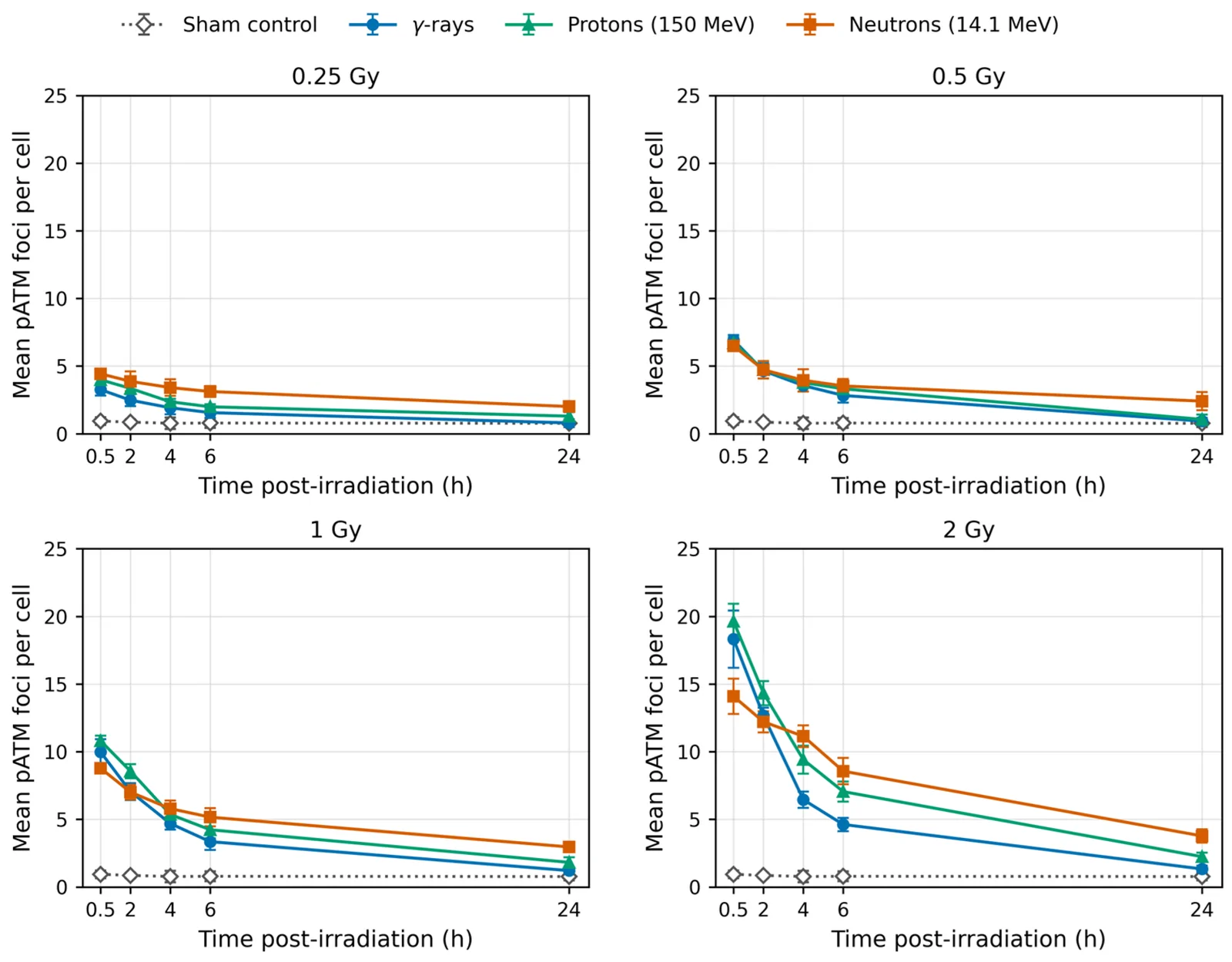
Experimentally measured kinetics of pATM foci in hMSCs after irradiation with γ-rays, 150 MeV protons, and 14.1 MeV neutrons at doses of 0.25, 0.5, 1.0, and 2.0 Gy. A common time-matched sham-control series is shown in each panel because all irradiation modalities were examined within the same independent experimental runs. Points represent means from three independent experiments (*n* = 3), and error bars represent standard errors calculated across the independent experimental means. Lines connect experimental means and are shown only to guide the eye.

We analyzed γH2AX and pATM foci kinetics after exposure to γ-rays, 150 MeV protons, and 14.1 MeV neutrons at doses of 0.25, 0.5, 1.0, and 2.0 Gy. Both markers were elevated at the first measurement at 0.5 h post-irradiation and subsequently declined over the observation period. The experimental time courses differed in the rate of signal resolution and in the magnitude of the residual signal at 24 h (Figure 1 and Figure 2).

Inspection of the experimental mean values showed no consistent ordering among γ-rays, protons, and neutrons at 0.5 h. During the subsequent decline, however, a clearer radiation-type-dependent pattern became apparent. At 24 h, the mean residual γH2AX and pATM signals were generally lowest after γ-irradiation, intermediate after proton irradiation, and highest after neutron irradiation.

Thus, radiation-type differences in γH2AX and pATM foci were reflected less in early response amplitude than in signal decay rate and late persistence.

**Figure 3:**
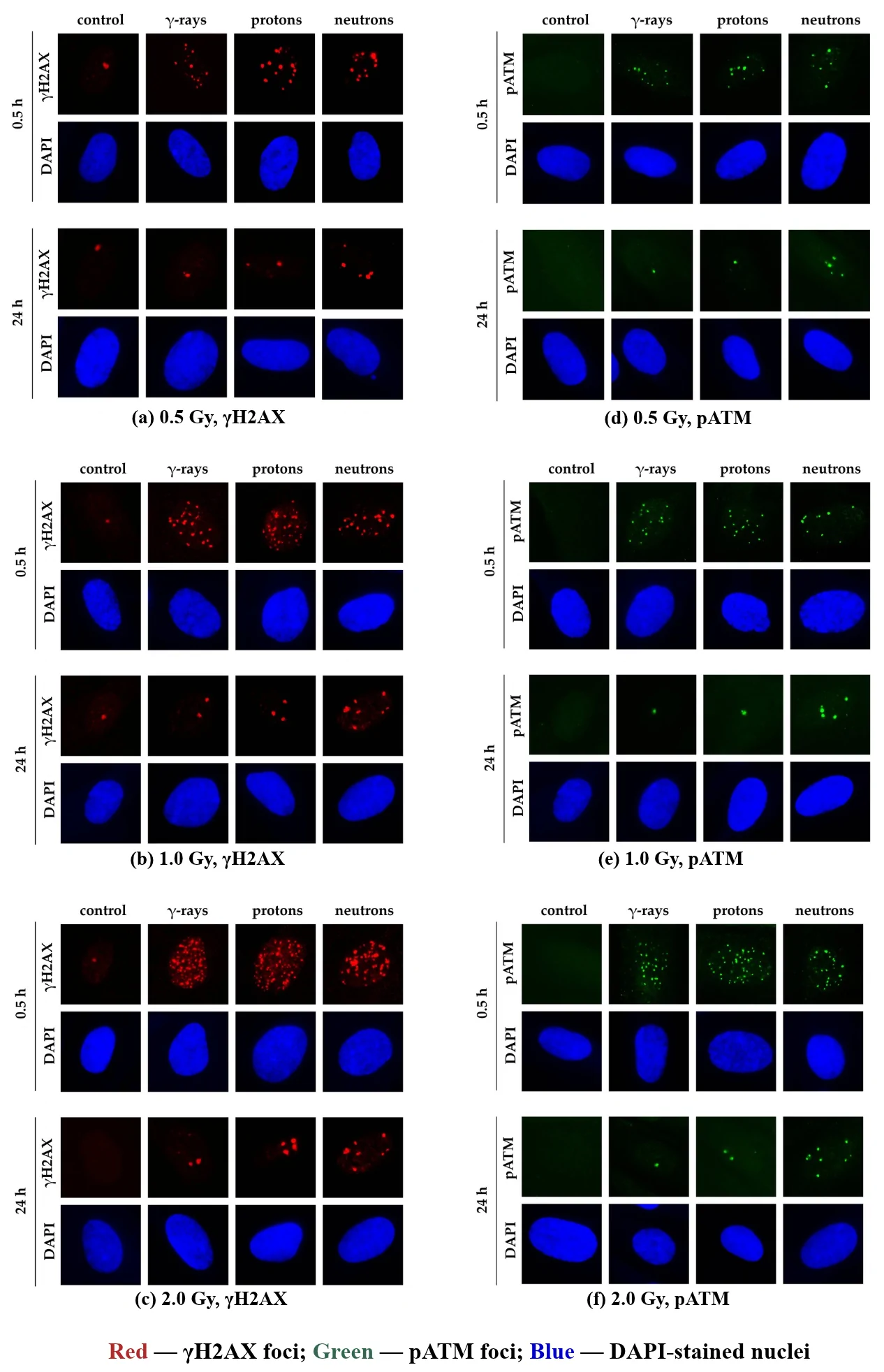
Representative immunofluorescence micrographs of hMSCs at 0.5 and 24 h after sham treatment or irradiation with γ-rays, 150 MeV protons, and 14.1 MeV neutrons (60× objective). Panels (**a**–**c**) show γH2AX staining at 0.5, 1.0, and 2.0 Gy, respectively, and panels (**d**–**f**) show pATM staining at the corresponding doses. γH2AX foci are shown in red, pATM foci in green, and nuclei counterstained with DAPI in blue. Representative images are shown for the earliest and latest experimental time points (0.5 and 24 h) to illustrate the early and late phases of the response; quantitative analysis included all measured time points.

Representative immunofluorescence micrographs illustrating γH2AX and pATM foci together with nuclear DAPI staining are shown in Figure 3.

**Figure 4:**
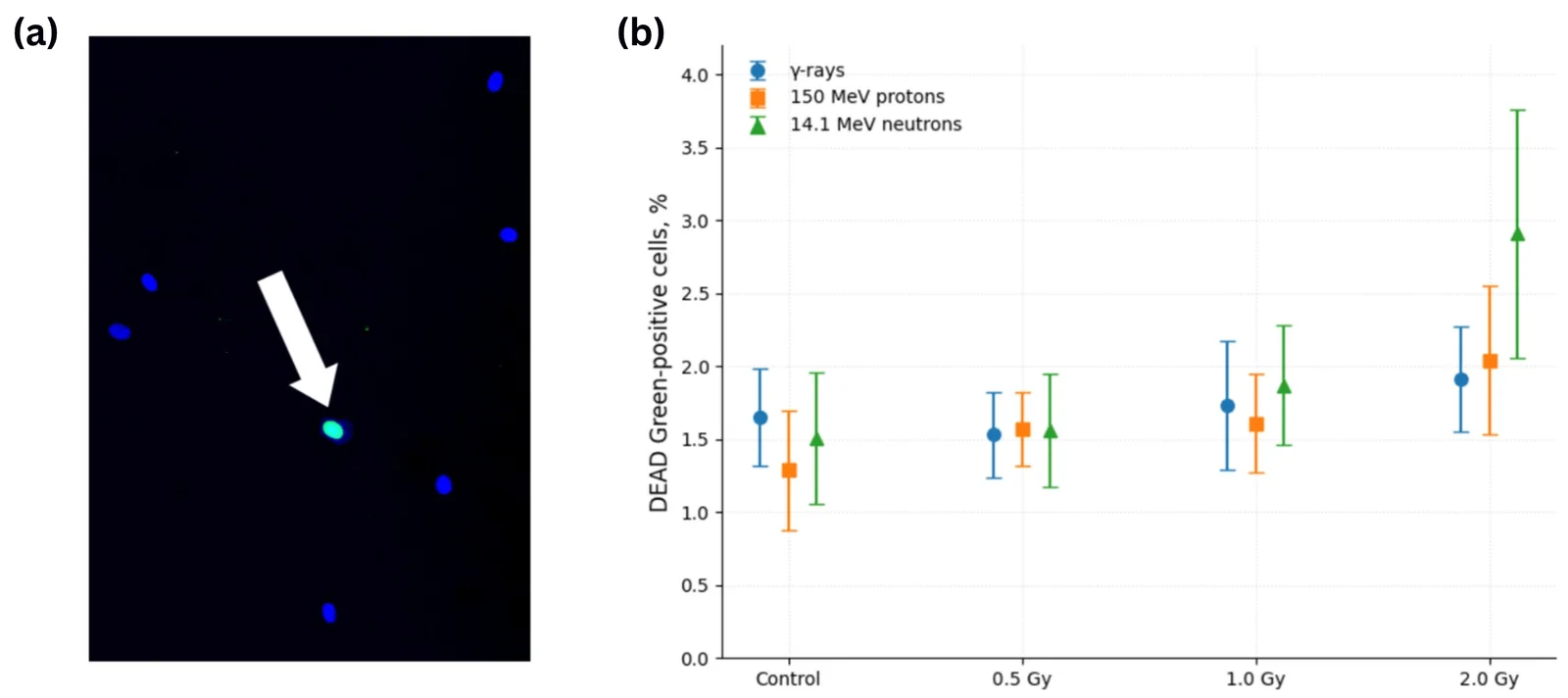
Cytotoxicity assessment in hMSCs 24 h after irradiation. (**a**) Representative fluorescence image of hMSCs stained with Image-iT™ DEAD Green™ Viability Stain; the arrow indicates a DEAD Green-positive nucleus of a cell with compromised plasma-membrane integrity (10× objective). (**b**) Percentage of DEAD Green-positive cells after exposure to γ-rays, 150 MeV protons, and 14.1 MeV neutrons at doses of 0.5, 1.0, and 2.0 Gy. Mean values derived from at least three independent experiments are shown. Error bars show SE.

To assess whether the observed DDR-marker persistence was accompanied by substantial cytotoxicity, cell membrane integrity was additionally evaluated 24 h after irradiation using DEAD Green staining under comparable irradiation conditions. The proportion of DEAD Green-positive cells remained low after γ-ray, 150 MeV proton, and 14.1 MeV neutron irradiation at 0.5, 1.0, and 2.0 Gy (Figure 4). Mean values remained low across all investigated conditions and did not exceed 3%. Although the cells irradiated with neutrons at a dose of 2 Gy demonstrated a trend towards an increase in the DEAD Green-positive cell fraction, this observation was not statistically significant (Figure 4).

### 2.2. Two-Exponential Approximation of Experimental Kinetics

Before constructing the kinetic model, we fitted the γH2AX and pATM time series with a biexponential function. This baseline analysis tested whether two characteristic timescales could reproduce the experimental curves without interpreting the fitted parameters as specific biological lesion fractions. Since the approximation was performed after subtraction of the control level, the model was specified as follows:

(1) $$y_{D}\left(t\right)=\;A_{\mathrm{fast}}\left(d\right)\exp\left(-\;\frac{∆t}{\tau_{\mathrm{fast}}}\right)+\;A_{\mathrm{slow}}\left(d\right)\exp\left(-\;\frac{∆t}{\tau_{\mathrm{slow}}}\right),$$

where Δ*t* = *t* − 0.5 h, and *A_fast_*(*d*) and *A_slow_*(*d*) are the amplitudes of the fast and slow components at dose *d*, while *τ_f_* and *τ_s_* are the corresponding characteristic times. The amplitudes *A_fast_*(*d*) and *A_slow_*(*d*) were estimated separately for each dose, whereas *τ_fast_* and *τ_slow_* were assumed to be common for a given marker and radiation type.

**Figure 5:**
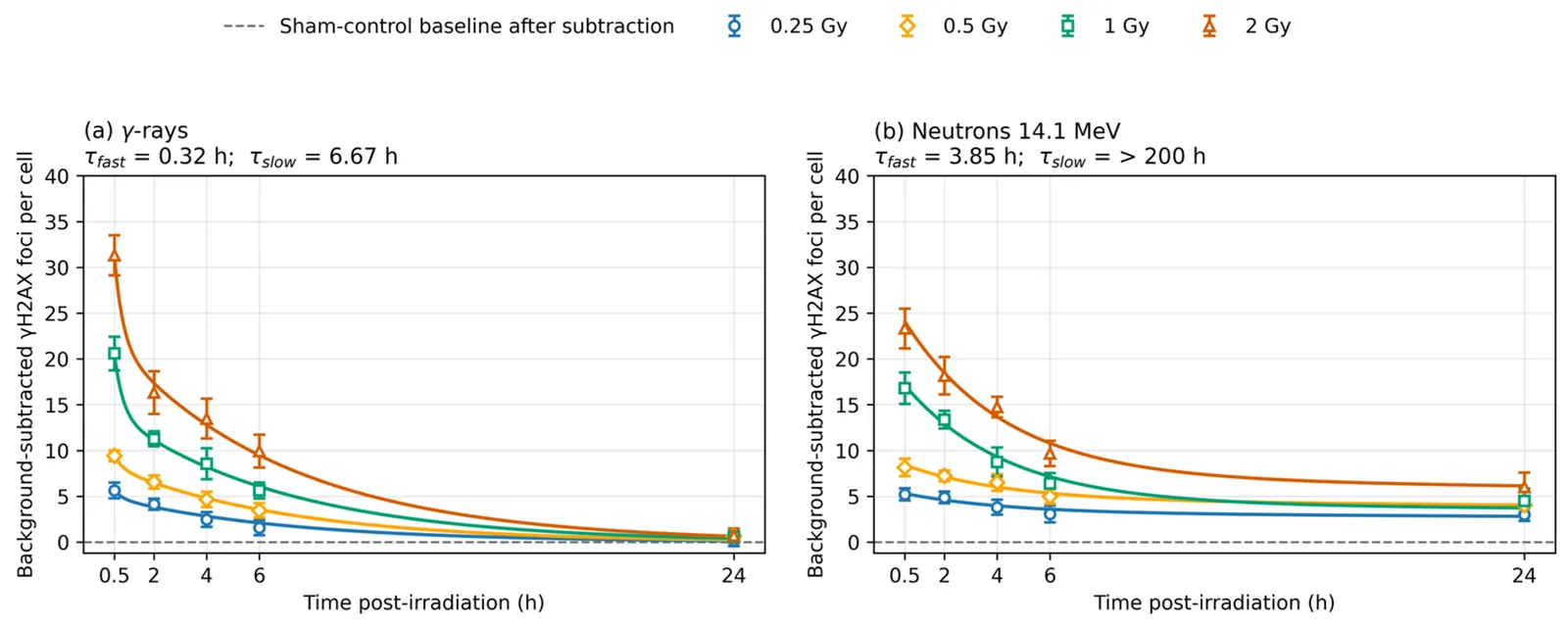
Double-exponential approximation of γH2AX foci kinetics after background subtraction: (**a**) γ-irradiation; (**b**) irradiation with 14.1 MeV neutrons. Points represent experimental mean values, error bars indicate standard errors, and lines show the fitted double-exponential curves. For each radiation type, the characteristic times *τ_fast_* and *τ_slow_* were shared across doses, whereas the amplitudes of the fast and slow components were estimated separately for each dose.

**Figure 6:**
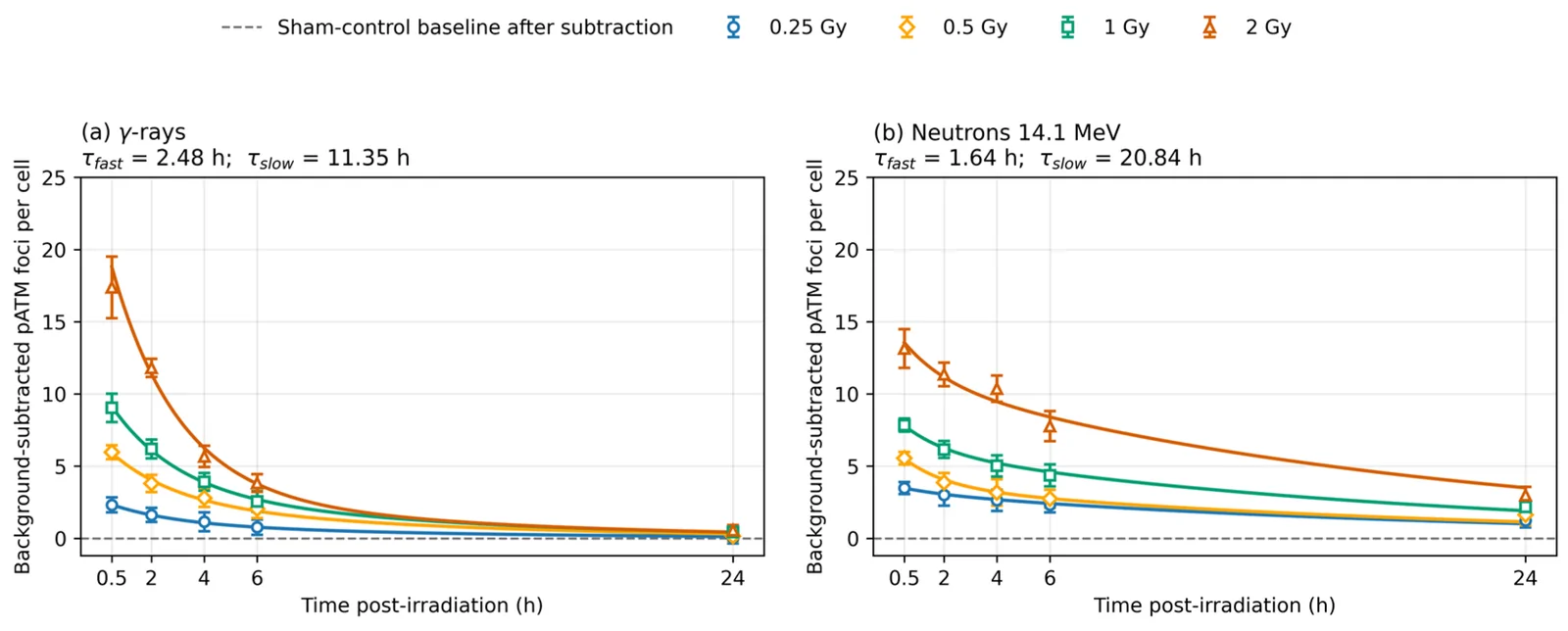
Double-exponential approximation of pATM foci kinetics after background subtraction: (**a**) γ-irradiation; (**b**) irradiation with 14.1 MeV neutrons. Points represent experimental mean values, error bars indicate standard errors, and lines show the fitted double-exponential curves. For each radiation type, the characteristic times *τ_fast_* and *τ_slow_* were shared across doses, whereas the amplitudes of the fast and slow components were estimated separately for each dose.

The fitting results are shown in Figure 5 and Figure 6, and the parameter values are listed in Table 1.

The two-exponential approximation faithfully reproduced the overall shape of the γH2AX and pATM time series. However, for γH2AX after neutron irradiation, the slow component was poorly constrained at the upper end within the 24-h observation interval: the estimate of *τ_s_* reached the upper bound of the allowable range. This indicates the presence of a long-lasting residual signal that cannot be reliably characterized solely in terms of a conventional slow exponential.

The two-exponential approximation reproduced the overall decay pattern of γH2AX and pATM signals after γ-ray and neutron irradiation. For γH2AX, the differences between radiation types were the most pronounced. After γ-irradiation, the characteristic times were *τ_fast_* = 0.32 h and *τ_slow_* = 6.67 h. After neutron irradiation, the fast component exhibited a longer time constant *τ_fast_* = 3.85 h, while the slow component reached the upper bound of the allowable parameter range. Consequently, the value *τ_slow_* > 200 h should not be interpreted as a directly measured repair half-time; rather, it reflects the presence of an almost non-decaying residual signal within the 24-h observation window. For pATM, the differences between radiation types were less pronounced but followed the same overall trend: the slow component after neutron irradiation was characterized by a longer decay time than after γ-irradiation. Overall, the two-exponential approximation points to the presence of a long-lasting component of the post-irradiation response, particularly pronounced for γH2AX after neutron exposure.

Because the two-exponential parameters are descriptive and cannot be assigned directly to specific biological lesion fractions, we next developed a kinetic model that partitions radiation-induced damage into effective repair components linked to pATM activation and γH2AX focus formation. In all kinetic models, radiation-induced damage is divided into a fast-repairing fraction, denoted *D_s_*, a complex slowly-repairing fraction *D_c_*, and—in the extended version—an effective ultraslow fraction *D_u_*. These damage-associated components collectively generate the ATM activation signal, with the contribution of the complex and ultraslow fractions to this signal governed by weight coefficients *w_dc_* and *w_du_*, respectively. The initial distribution of damage is described by the fraction of the fast component *α* and the fraction of the ultraslow component within the slow pool *π*. A complete description of the model is provided in the Materials and Methods section.

### 2.3. Two-Component Model Reproduces the Data but Conceals the Late Component Within the Complex Fraction

We first asked whether a minimal two-component scheme—partitioning damage only into a fast-repairing fraction D*_s_* and a complex, saturably repaired fraction *D_c_*—could describe the data. When fitted independently at each dose to the γH2AX and pATM data jointly, the model captured the overall curve shapes with acceptable residuals (Table 2).

**Figure 7:**
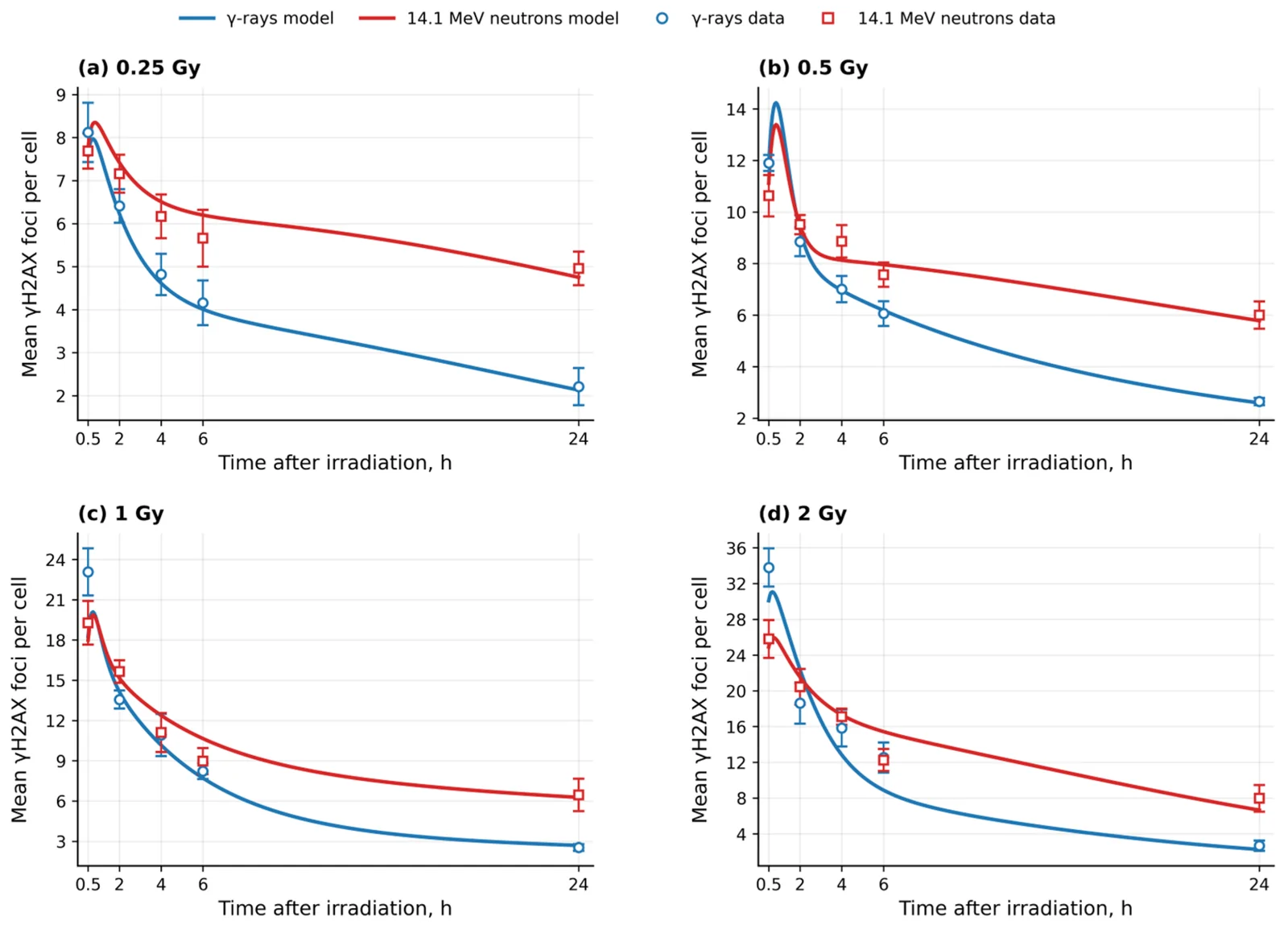
Two-component baseline model for γH2AX kinetics. Experimental γH2AX foci kinetics after γ-ray and 14.1 MeV neutron irradiation are shown together with two-component model predictions fitted independently for each dose (0.25 Gy (**a**), 0.5 Gy (**b**), 1.0 Gy (**c**) and 2.0 Gy (**d**)). The model captures the overall decay pattern, while the higher residual signal after neutron irradiation is represented through the effective complex-damage component.

**Figure 8:**
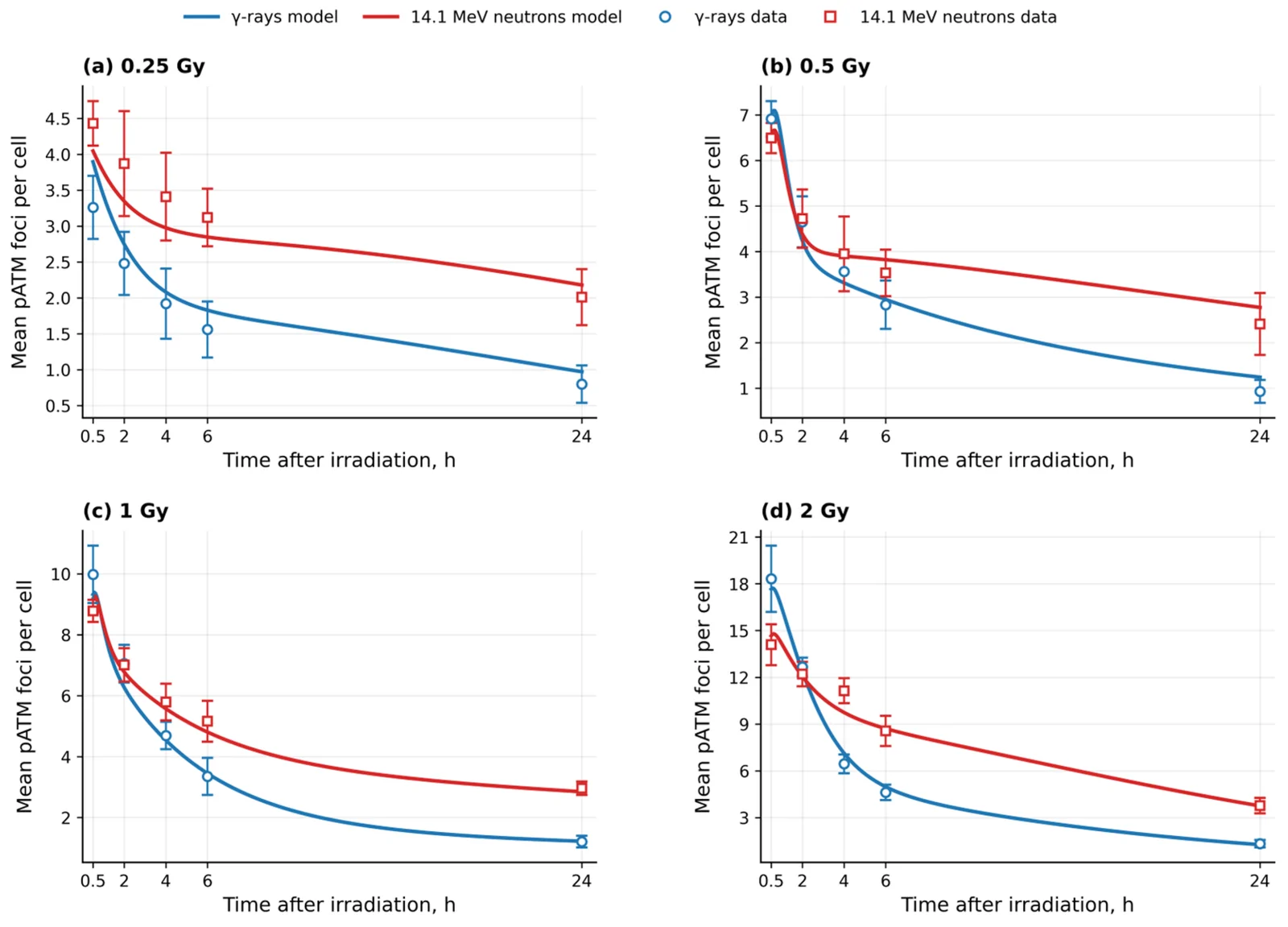
Two-component baseline model for pATM kinetics. Experimental pATM foci kinetics after γ-ray and 14.1 MeV neutron irradiation are shown together with two-component model predictions fitted independently for each dose (0.25 Gy (**a**), 0.5 Gy (**b**), 1.0 Gy (**c**) and 2.0 Gy (**d**)). As for γH2AX, the elevated late neutron response is absorbed into the effective complex-damage component rather than represented as a separate late-persistent fraction.

How the model achieved this fit was informative. To reproduce the elevated late neutron signal, it assigned a larger complex fraction to neutrons than to γ-rays at most doses and gave that fraction greater effective weight in the ATM activation signal (Table 2). Thus, removing the ultraslow fraction did not eliminate the radiation-quality difference; it redistributed that difference to the size and weight of the slow fraction. The model also showed systematic local deviations, most consistently underestimating γH2AX after high-dose γ-irradiation. This suggests that a single slow fraction cannot capture both an intermediate plateau and the subsequent rapid decline of the γ-response, motivating explicit inclusion of a late component (Figure 7 and Figure 8).

### 2.4. Effective Ultraslow Component Provides a Model-Based Index of Late DDR Persistence

We therefore introduced an effective ultraslow fraction *D_u_*, defined not as a separate molecular class of lesions, but as a kinetic component required to describe the long-lasting residual signal. The normalized initial fraction of the ultraslow component, *D_u_*_0_/*N*_0_ = (1 − *α*)*π*, was used as the primary descriptor of radiation quality. This quantity, rather than *D_c_*_0_/*N*_0_, was chosen as the main index of late repair burden, because *D_c_* in the model represents a broad pool of complex lesions involved in both early and intermediate phases of the response; this parameter is sensitive to the initial partitioning of damage, saturable repair kinetics, and the weight of the component in the observed DDR signal. By contrast, *D_u_* was introduced specifically as an effective component associated with late signal persistence. Hence, *D_u_*_0_/*N*_0_ is interpreted not as the true fraction of complex lesions, but as a model-based index of late damage-associated DDR load.

**Figure 9:**
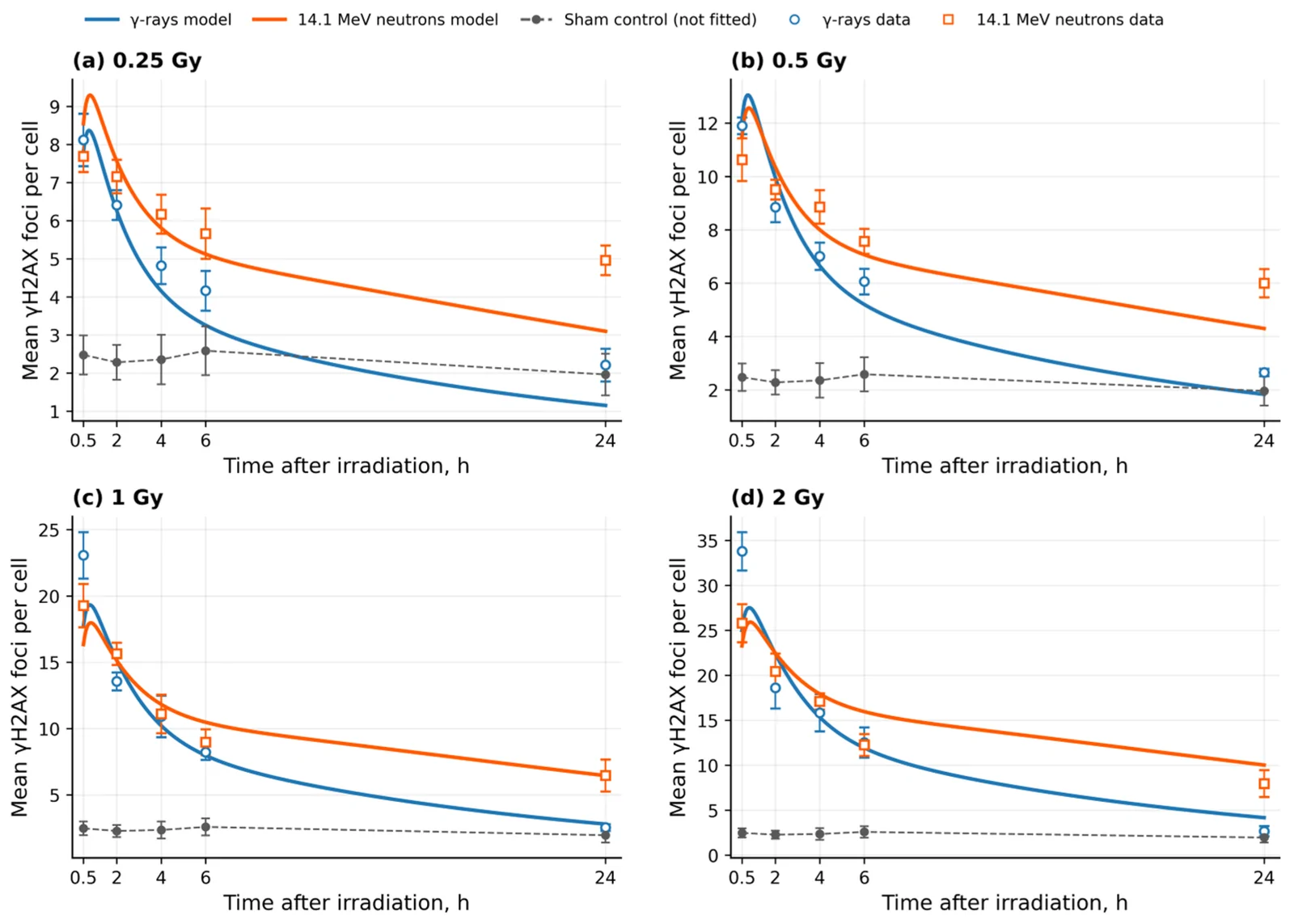
Extended (three-component) model predictions versus experimental data for γH2AX across all doses (0.25 Gy (**a**), 0.5 Gy (**b**), 1.0 Gy (**c**), and 2.0 Gy (**d**)), grouped by radiation type, for the selected tied-weight model. Points represent experimental means (±standard error), and solid lines show the model fit; blue denotes γ-rays and red denotes 14.1 MeV neutrons. The model captures the overall early response and the elevated late signal after neutron irradiation across the investigated dose range.

**Figure 10:**
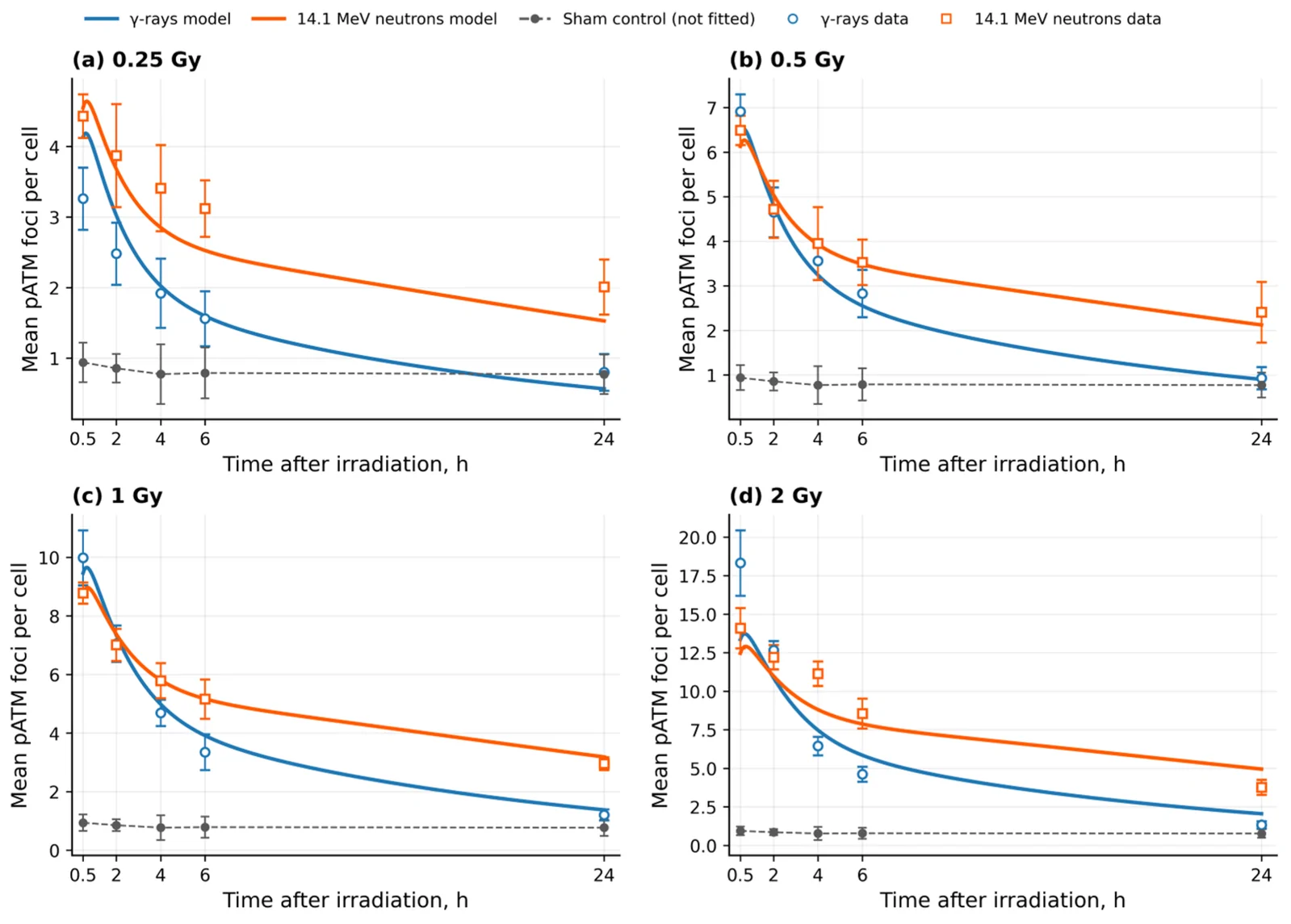
Extended (three-component) model predictions versus experimental data for pATM across all doses (0.25 Gy (**a**), 0.5 Gy (**b**), 1.0 Gy (**c**), and 2.0 Gy (**d**)), grouped by radiation type, for the selected tied-weight model. Points represent experimental means (±standard error), and solid lines show the model fit; blue denotes γ-rays and red denotes 14.1 MeV neutrons. The model captures the overall early response and the elevated late signal after neutron irradiation across the investigated dose range.

**Figure 11:**
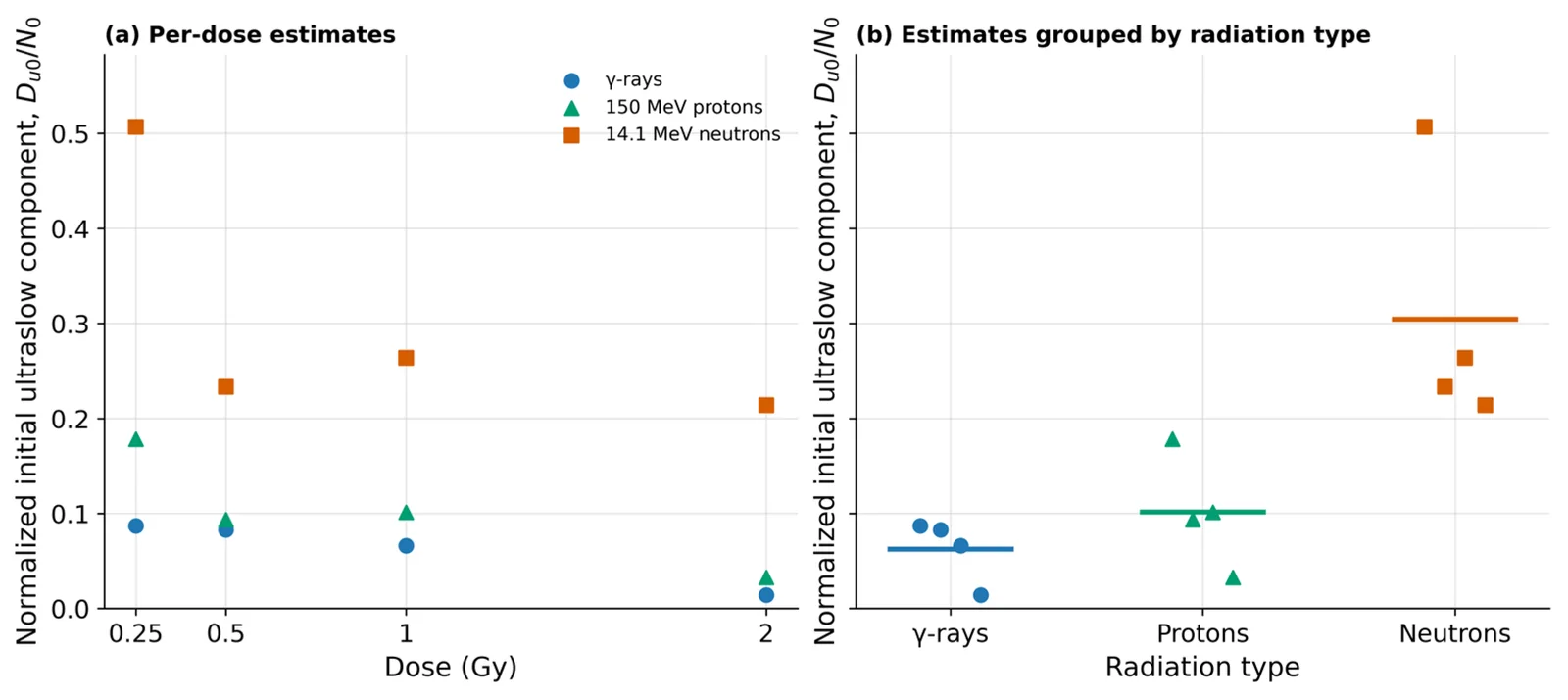
Normalized ultraslow fraction *D_u_*_0_/*N*_0_ by radiation type and dose (per-dose estimates). (**a**) At each dose, the values followed the order γ-rays < protons < neutrons. (**b**) The values clustered by radiation type, with the spread between radiation types exceeding the spread across doses within a given type, consistent with a stronger association of the late-persistent DDR index with radiation quality than with dose. Horizontal bars denote the mean across doses for each radiation type. The γ–neutron separation was supported by parametric bootstrapping (P(neutrons > γ) = 0.97; see Table 4).

With per-dose fitting to the data for γ-rays, protons and neutrons, the model placed the ultraslow fraction in a strict order γ < protons < neutrons at each dose, without exception (Table 3). Protons were consistently intermediate, but did not occupy a fixed midpoint: at low doses, proton values lay notably below neutron values, whereas at higher doses they remained closer to the γ-ray end of the range, consistent with their lower average ionization density compared to 14.1 MeV neutrons (Figure 9 and Figure 10; the ordering is summarized in Figure 11).

**Table 4:** Bootstrap estimates of the normalized ultraslow fraction *D_u_*_0_/*N*_0_ (100 replicates, pooled scheme). The wide confidence intervals reflect the multimodality of the likelihood surface and the non-identifiability of the absolute values; the robust outcome is the γ–neutron separation, expressed through P(neutrons > γ).

Radiation Type	Median *D_u_*_0_/*N*_0_	95% CI
γ-rays	0.027	0.002–0.166
Neutrons 14.1 MeV	0.434	0.061–0.671
P(neutrons > γ)	0.97	—

### 2.5. Pooled Fitting and Model Comparison: The Ordering Is Robust, but Absolute Fractions Are Not Identifiable

To test whether the ordering is preserved when kinetic and signaling parameters are tied across doses, we performed a pooled fitting procedure in which the dose dependence enters solely through *N*_0_(*d*). The introduction of the ultraslow fraction reduced the χ^2^ value relative to the two-component baseline model, as shown in Table 5, indicating a lower joint residual error relative to the two-component baseline model.

At the same time, the pooled analysis revealed that the absolute ultraslow fractions are not uniquely identifiable. The likelihood surface is multimodal: several local minima within Δχ^2^ ≈ 20 of the global minimum yielded different absolute values of *D_u_*_0_/*N*_0_, and some of these basins were inconsistent in terms of the ordering. In a diagnostic model with a free weight of the ultraslow fraction, denoted *w_du_*, varied within the constraint *w_du_* ≤ 1, which formally achieved the lowest AICc, this manifested as a weight-fraction degeneracy: a small specific weight was compensated by a large initial fraction, meaning that a low *w_du_* combined with a high *π* produced nearly the same signal as a high *w_du_* with a low *π*, resulting in absolute values that cannot be interpreted literally. Tying the ultraslow weight to the weight of the complex fraction, i.e., setting *w_du_* = *w_dc_*, eliminated this degeneracy and was therefore chosen as the primary model structure, even though it did not yield the lowest AICc; model selection was based on the identifiability of the target quantity rather than on AICc alone.

The flexible model with an explicit late component gave the lowest AICc, whereas the model with tied weights was employed as an interpretable diagnostic framework to verify whether the ordering among radiation types is preserved when the degeneracy between the weight and the initial fraction of the late component is removed.

For these reasons, pooled fitting was used only to verify the robustness of the ordering, whereas quantitative estimates of *D_u_*_0_/*N*_0_ were taken from per-dose fits. Despite the dependence of the absolute pooled values on the optimization basin, the separation between γ-rays and neutrons was preserved in the primary tied-weight model and was supported by parametric bootstrapping. Thus, the robust and interpretable result is the ranking by radiation quality, rather than the absolute magnitude of the ultraslow fraction.

**Table 5:** Model comparison in the pooled scheme (joint fitting of γH2AX and pATM for γ-rays and neutrons across all doses; n = 80). AICc denotes the Akaike information criterion corrected for finite sample size; ΔAICc is relative to the lowest value. Although the diagnostic model with a free ultraslow weight attains the lowest AICc, its absolute ultraslow-fraction estimates are degenerate; the tied-weight model was therefore retained as the primary structure.

Model	Description	k	df	χ^2^	AIC	AICc	ΔAICc
Two-component	baseline, without ultraslow fraction	21	59	240.6	282.6	298.6	7.6
Main (*w_du_* = *w_dc_*)	*π* is free, *k_cu_* is free	25	55	234.5	284.5	308.6	17.6
Diagnostic (free *w_du_* ≤ 1)	independent weight of the ultraslow fraction	26	54	212.5	264.5	291.0	0

### 2.6. Within the Investigated Dose Range, Late RAD51 Persistence Is More Strongly Associated with Radiation Quality than with Dose

**Figure 12:**
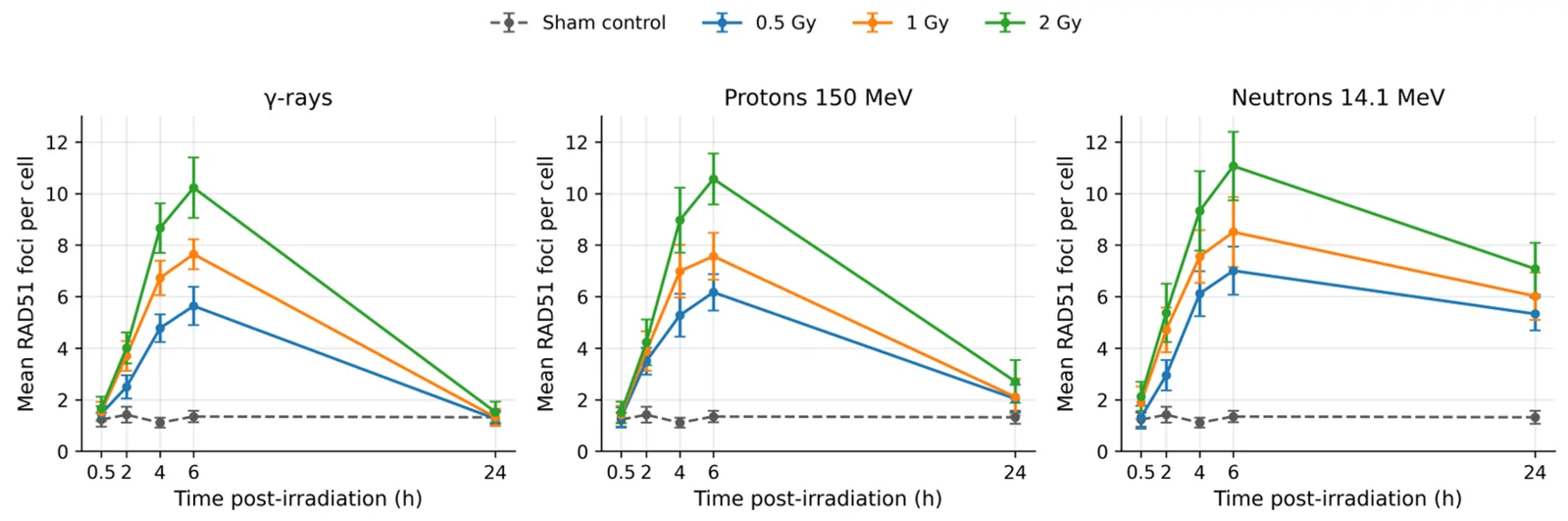
Dynamics of RAD51 after γ-ray, proton, and neutron irradiation at doses of 0.5 Gy, 1.0 Gy, and 2.0 Gy.

**Figure 13:**
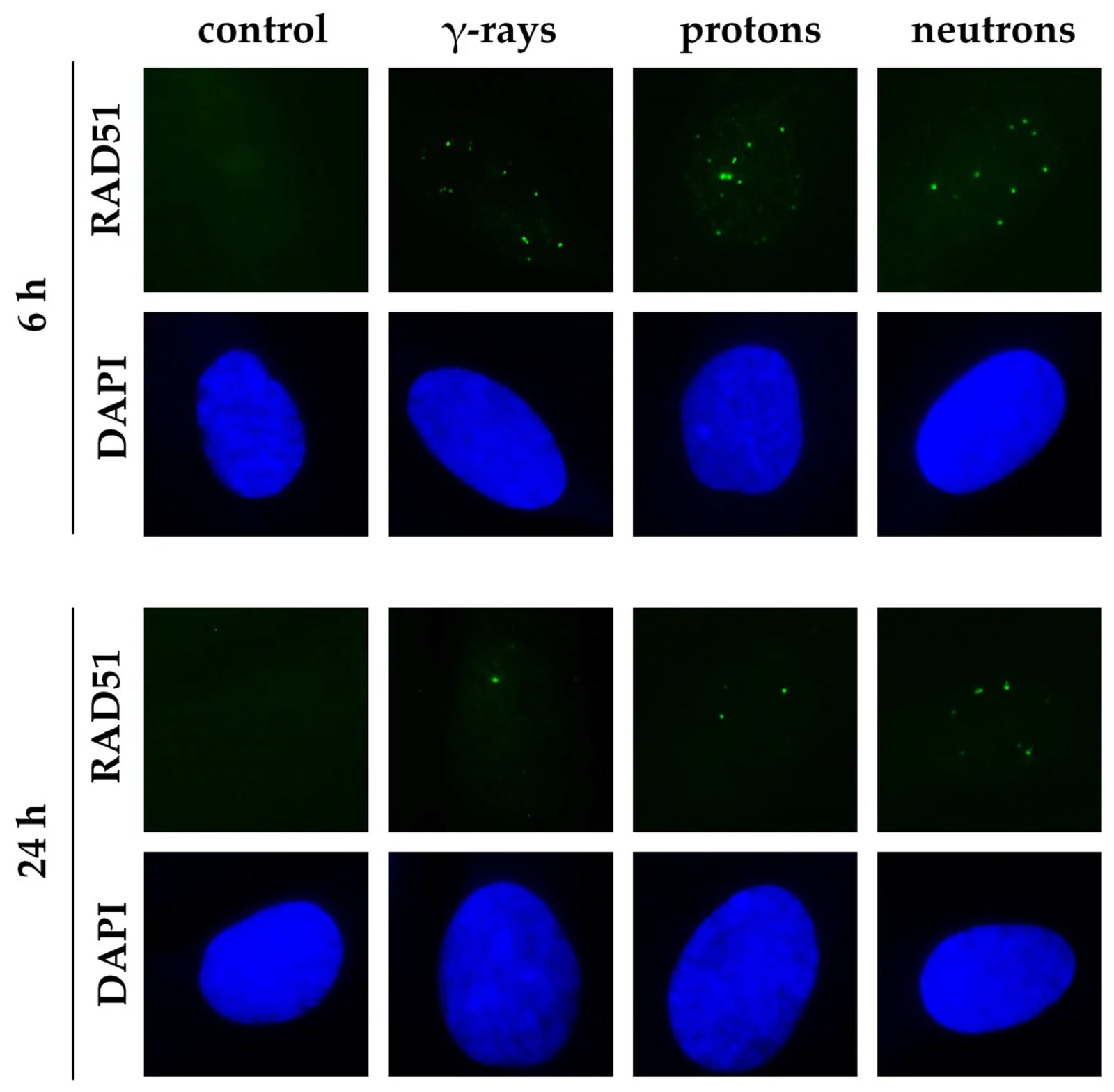
Representative immunofluorescence micrographs of hMSCs at 6 and 24 h after sham treatment or irradiation with γ-rays, 150 MeV protons, and 14.1 MeV neutrons at a dose of 1 Gy (60× objective). RAD51 foci are shown in green. Nuclei counterstained with DAPI are shown in blue.

To assess whether the observed ordering was associated more strongly with radiation quality than with dose, we analyzed variation in the residual RAD51 signal at 24 h post-irradiation. RAD51 was not used in model fitting and was therefore treated as a complementary biological readout. The complete RAD51 time courses are shown in Figure 12. Representative RAD51 immunofluorescence micrographs are shown in Figure 13.

**Figure 14:**
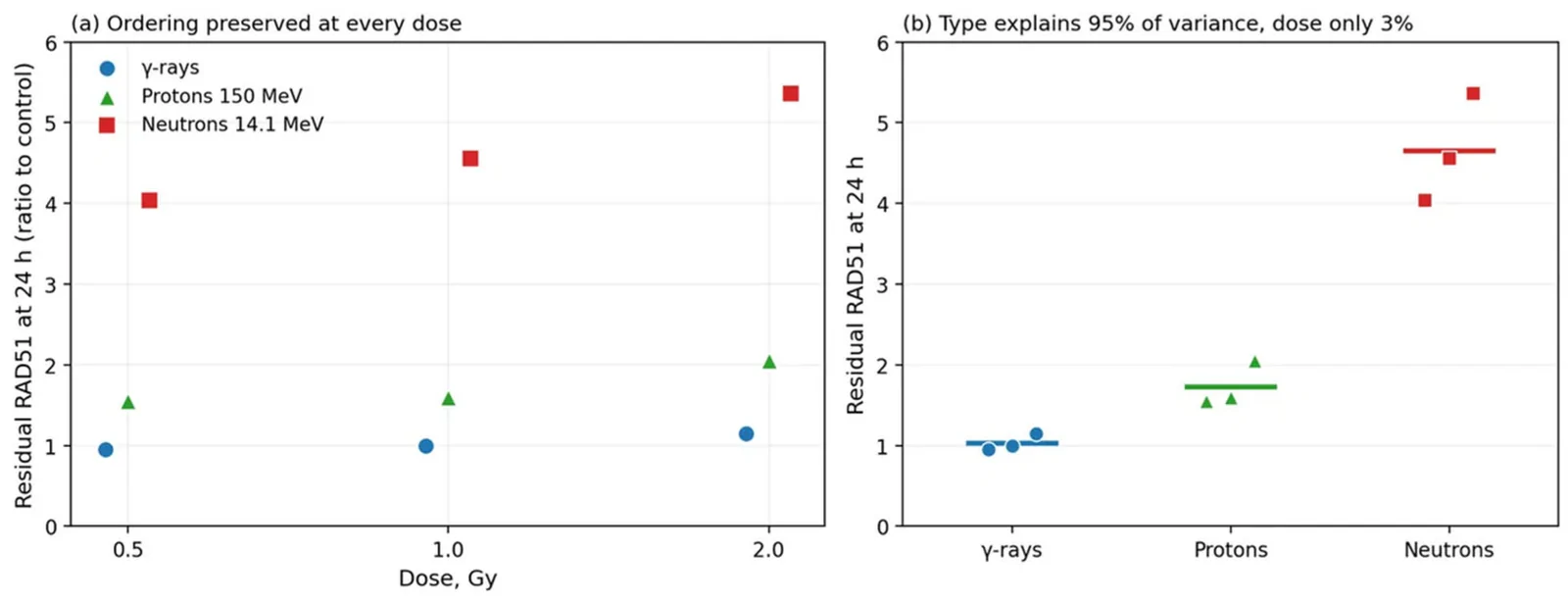
Residual RAD51 signal at 24 h post-irradiation, normalized to the corresponding time-matched sham control, by radiation type and dose. RAD51 was not used in model fitting and is shown as a complementary biological readout. (**a**) At every investigated dose, the values followed the order γ-rays < protons < neutrons. (**b**) The values clustered by radiation type; in the descriptive two-way additive sum-of-squares decomposition, radiation type accounted for 95.4% of the total variation and dose accounted for 3.1%. Horizontal bars denote the mean across doses for each radiation type.

Within the investigated dataset, residual RAD51 at 24 h varied primarily among radiation types rather than among doses. In the descriptive two-way additive sum-of-squares decomposition, radiation type accounted for 95.4% of the total variation, dose accounted for 3.1%, and the remaining 1.5% represented residual variation, including any interaction that could not be estimated separately (Table 6). Within each dose, neutrons produced a 4.3- to 4.7-fold higher residual RAD51 signal than γ-rays, whereas variation across doses within a given radiation type did not exceed a factor of 1.2–1.3 (Table 7). This pattern is consistent with the model-derived normalized late-persistent index *D_u_*_0_/*N*_0_, which related to specific radiation types but did not show a systematic dose-dependent trend (Figure 11 and Figure 14).

**Table 6:** Descriptive two-way additive sum-of-squares decomposition of the residual RAD51 signal at 24 h post-irradiation (0.5–2.0 Gy).

Source of Variation	Percentage of Total Variance
Radiation type	95.4
Dose	3.1
Residual variation	1.5

Taken together, these results show that, within the investigated dose range and experimental system, variation in late RAD51 foci persistence was associated more with radiation type than with absorbed dose. The concordant ordering of the normalized ultraslow index, the residual RAD51 signal, and the track-structure-based expectations supports the interpretation of the ultraslow component as a comparative model-based descriptor of late-persistent DDR signaling. It does not, however, establish that this component represents a specific physical lesion class or that its absolute value is independent of model parameterization.

**Table 7:** Residual RAD51 signal at 24 h post-irradiation (ratio to control) as a function of radiation type and dose (0.5–2.0 Gy).

Radiation Type	0.5 Gy	1.0 Gy	2.0 Gy	Mean
γ-radiation	0.95	1.00	1.15	1.03
150 MeV protons	1.55	1.60	2.05	1.73
14.1 MeV neutrons	4.04	4.56	5.36	4.65

## 3. Discussion

The principal finding of the present study is that, within the investigated hMSC system and dose range, radiation quality was more associated with late DDR-marker persistence than absorbed dose. At the earliest experimental time point (0.5 h), γH2AX and pATM foci counts increased with dose but showed no consistent ordering among radiation types. Differences became more apparent during signal resolution and at 24 h, when the residual γH2AX and pATM signals followed the order γ-rays < protons < neutrons at each investigated dose. This pattern was supported by kinetic modeling, complementary RAD51 measurements, and the expected differences in track structure among the three radiation qualities.

The biexponential approximation provided an initial descriptive indication that the γH2AX and pATM time courses contained more than one kinetic timescale. However, its fitted exponential components were not interpreted as distinct molecular classes or direct fractions of DNA damage. The extended effective kinetic model provided a structured representation of the same multiphasic behavior and allowed late persistence to be summarized by the normalized initial ultraslow component, *D_u_*_0_/*N*_0_. In the per-dose fits, this model-derived index followed the order γ-rays < protons < neutrons at all four doses (Table 3). Its absolute values remained dependent on model structure and were only partially identifiable in the pooled analysis. Nevertheless, in the parametric bootstrap of the pooled γ-ray and neutron data, the neutron value exceeded the γ-ray value in 97% of replicates (Table 4). The robust result is therefore the radiation-quality ordering of the index, rather than its absolute numerical magnitude or its interpretation as a literal lesion fraction.

RAD51 provided a complementary biological readout because it was analyzed separately and was not used for parameter estimation in the γH2AX/pATM models. At 24 h, the RAD51 signal normalized to the corresponding sham control followed the same qualitative order, γ-rays < protons < neutrons, at each investigated dose. In the descriptive two-way additive sum-of-squares decomposition, radiation type accounted for 95.4% of the total variation in the observed radiation-type × dose matrix, whereas dose accounted for 3.1% (Table 6). Because only one aggregated value was available for each combination, these percentages do not constitute an inferential analysis of variance, and the interaction between radiation type and dose could not be estimated separately. RAD51 participates in homologous recombination, and its late persistence is therefore consistent with prolonged repair-associated activity [37,38]. However, RAD51 is predominantly active in S/G2-phase cells, and the experiment did not include cell-cycle-phase resolution. Differences in the residual RAD51 signal may consequently reflect differences in cell-cycle distribution as well as differences in damage processing. RAD51 should therefore be regarded as supportive complementary evidence rather than as independent mechanistic validation of the model-derived ultraslow component.

Physical track-structure considerations provide a plausible context for the observed radiation-quality dependence. Compared with sparsely ionizing γ-rays, higher-LET radiation produces more spatially concentrated energy deposition and is more likely to generate clustered DNA damage, including DSBs accompanied by nearby base damage, single-strand breaks, or chemically modified DNA ends. Such lesions may require additional processing before repair can be completed and may contribute to prolonged DDR-marker persistence. In the present comparison, 150 MeV protons showed an intermediate late response, whereas 14.1 MeV neutrons, which generate densely ionizing secondary charged particles in biological matter, produced the highest residual γH2AX, pATM, and RAD51 signals. These physical considerations are consistent with, but do not by themselves prove, the proposed relationship between radiation quality and late DDR persistence [16,17,18,19,20,21,23,24].

The concordance among the late γH2AX and pATM signals, the model-derived ultraslow index, and the complementary RAD51 measurements supports the presence of a radiation-quality-dependent difference in late DDR persistence. In this study, the term “late repair burden” does not denote a directly measured number of unrepaired DSBs. It is used as an operational, model-based description of the magnitude and persistence of damage-associated signaling remaining at late post-irradiation times. Because γH2AX, pATM, and RAD51 foci reflect several stages of damage recognition, signaling, chromatin modification, and repair-factor recruitment, none of these markers can be interpreted as a one-to-one measure of a specific lesion class. Accordingly, the ultraslow component should be interpreted as a comparative index of late-persistent DDR signaling rather than as a literal fraction of complex or irreparable DNA lesions.

The observed ordering, γ-rays < protons < neutrons, is broadly consistent with increasing ionization density and with the approximate representative LET values used to characterize these radiation fields: 0.2 keV/µm for ^60^Co γ-rays, 0.5 keV/µm for 150 MeV protons, and 12 keV/µm for 14.1 MeV neutrons. In this work, radiation type was treated as a categorical factor rather than as an explicit numerical LET variable; LET is therefore invoked to interpret the direction of the effect rather than as a predictor in the model. The relatively low LET of 150 MeV protons in the plateau region is consistent with their proximity to the γ-ray end of the range. An explicit treatment of LET as a continuous variable, together with track-structure modeling, will be a subject of future work.

In particular, we examined the hypothesis of limited throughput capacity of the repair system, according to which late signal persistence could arise because the number of lesions exceeds the available repair complexes, causing some lesions to queue for processing. However, implementation of this hypothesis as a Markov process with a finite number of processing units did not reproduce the observed late kinetics within a physically reasonable parameter range: the queue dissipated and the characteristic persistent tail did not emerge. Thus, within the tested formulation, limited repair capacity alone was insufficient to account for the late signal.

This result motivated the use of *D_u_* as an explicit effective descriptor of late-persistent DDR signaling rather than as a mechanistically identified lesion class. The emerging picture is consistent with current views on the spatial organization of double-strand break repair. Damage-containing chromatin domains may undergo spatial clustering during repair [14,39], and under densely ionizing irradiation such organization could contribute to prolonged retention of repair proteins and delayed signal resolution. The biological mechanism underlying the late component, however, remains unresolved and will require direct track-structure and spatial-damage modeling.

The results should be viewed in the context of existing knowledge on the influence of radiation quality on DNA repair. It is well established that densely ionizing radiation produces a higher proportion of complex lesions that may require prolonged processing, and that high LET is associated with prolonged marker persistence, as documented in numerous classical and recent studies [13,16,17,23]. In this sense, the mere fact of stronger late persistence after neutron irradiation is not novel. The contribution of the present work lies not in discovering this effect, but in comparative kinetic characterization and systematic analysis.

First, we compared the associations of radiation quality and absorbed dose with the late phase of the response. Previous studies have shown that radiation quality and LET are closely associated with lesion complexity and prolonged DDR-marker persistence [16,17,18,19,20,21,23,24]. In the present study, we directly compared the associations of radiation quality and absorbed dose with the late phase of the response within the same experimental system. In the descriptive two-way additive sum-of-squares decomposition of the normalized 24-h RAD51 values, radiation type accounted for approximately 95% of the total variation, whereas dose accounted for only a few percent. These percentages are descriptive and should not be interpreted as an inferential demonstration of causal dominance. With the γH2AX and pATM kinetics, this analysis supports a stronger association of radiation quality than dose with late DDR-marker persistence under the investigated conditions.

Second, we performed a systematic comparison of three radiation types (γ-rays, protons, and neutrons) in the same cell system, using identical markers and a unified analysis procedure. Notably, proton irradiation consistently produced an intermediate late response between γ-rays and neutrons. Thus, comparing three radiation qualities revealed an ordered pattern that would not be apparent from a two-group comparison. This within-system comparison, performed using the same cell model, DDR markers, time points, and analysis framework, is a distinct strength of the present study and reduces the methodological heterogeneity that can complicate comparisons across separate experiments.

Third, we paid particular attention to the identifiability and robustness of the model estimates. Multi-start analysis, bootstrapping, and comparison of several model structures showed that the absolute estimates of the ultraslow fraction in the pooled scheme are model-dependent, whereas the ranking by radiation quality was robust. Such an explicit analysis of the limits of identifiability of kinetic models is rarely performed in radiobiological repair modeling, where fitting parameters are often interpreted directly [39]. We believe that separating robust conclusions (the ranking) from non-robust ones (absolute fractions) is an important element of a correct interpretation of such models.

Thus, the advancement of the present work lies in the comparative quantitative characterization of late DDR-marker persistence across three radiation qualities within the same experimental system, together with the explicit distinction between the robust radiation-quality ranking and model-dependent absolute parameter estimates.

From an applied perspective, these findings indicate that absorbed dose alone may not fully characterize the duration of DDR-associated signaling in normal stromal cells when radiation qualities differ. In comparative radiobiology and radiation protection, kinetic endpoints may therefore complement absorbed-dose measurements when evaluating exposures involving different or mixed radiation fields. In radiotherapy, the observed differences among γ-rays, plateau-region protons, and the higher-LET neutron reference emphasize the potential value of considering the temporal resolution of normal-tissue DDR markers in addition to the delivered dose. However, the present in vitro marker-based results do not provide a direct estimate of clinical toxicity or relative biological effectiveness and should not be used alone for treatment planning or radiation-risk assessment.

### Limitations

Several limitations of the present study should be considered when interpreting the results. Although a total of 450 cells were analyzed per experimental condition across the three independent experiments, the reported standard errors and the experimental uncertainties used for model fitting were calculated from only three independent experimental means (*n* = 3). Treating the independent experiment, rather than individual cells or slides, as the biological replicate avoids pseudoreplication. However, the small number of independent experiments limits the precision of the estimated standard errors and goodness-of-fit comparisons. The conclusions therefore rely primarily on reproducible qualitative patterns, particularly the radiation-quality ordering of the late response, rather than on small differences in individual fitted parameter values.

The estimation of the ultraslow component relies mainly on the 24-h time point, since no measurements were taken between 6 and 24 h. Within the observation window, the slow and ultraslow components are only partially separated, and the absolute characteristic time of the ultraslow fraction is imprecisely determined. Additional intermediate time points, in particular, between 8 and 12 h, together with later measurements beyond 24 h, would allow a sharper separation of the slow and ultraslow components and are a subject of future work. Importantly, although the absolute parameter estimates remain uncertain, the qualitative ordering of the normalized ultraslow component by radiation type was preserved across all investigated doses and fitting schemes. Also, an additional modeling limitation is that finite irradiation duration was not explicitly represented. This approximation is expected to be most relevant to the earliest part of the response, particularly for neutron irradiation because of its lower dose rate.

The absolute values of the internal damage fractions in the pooled scheme are only partially identifiable. As shown above, the objective-function surface is multimodal, and in the model with a free ultraslow weight, the weight and the initial fraction compensate each other. Therefore, we consider the ranking of radiation types, rather than the literal fraction values, as the robust result. This limitation reflects the partial identifiability of effective kinetic models of this class and should be taken into account when interpreting fitted parameter values.

The proposed ultraslow component is an effective kinetic component and is not identified with a separate molecular class of lesions. Its biological interpretation (complex clustered lesions, chemically modified DNA ends, prolonged lesion processing, and chromatin-associated signaling) remains hypothetical and requires direct verification by track-structure modeling and analysis of spatial damage distribution. The concordance with the complementary RAD51 measurements supports the relevance of the radiation-quality-dependent late-persistence pattern but does not establish the molecular identity of the model-derived ultraslow component.

A further limitation is that the present study did not assess long-term survival or clonogenic capacity. Acute cytotoxicity was additionally evaluated by DEAD Green staining and remained low across the tested conditions, with mean values not exceeding 3% after γ-ray, 150 MeV proton, or 14.1 MeV neutron irradiation at 0.5–2.0 Gy. This finding is consistent with previous reports of relatively limited acute viability effects in mesenchymal stromal cells after moderate photon doses: viability after 2.5 Gy was comparable with that of controls in bone-marrow MSCs, while no statistically significant reduction in viability was detected after 5 Gy over a 24–168 h interval [40,41]. However, the DEAD Green assay reflects loss of plasma-membrane integrity and does not provide information on delayed cell death, reproductive survival, or clonogenic capacity. The residual DDR-marker signals therefore cannot be directly translated into long-term survival outcomes. Future studies should combine DDR-marker kinetics with complementary functional endpoints, including apoptosis, metabolic viability, clonogenic survival, and chromosomal-aberration assays.

Finally, the analysis was restricted to a single cell type and tissue source—human adipose-derived mesenchymal stromal/stem cells—and did not include cell-cycle-phase resolution, although cell-cycle status can influence γH2AX-based DNA damage assessment [42], while RAD51 formation is closely linked to homologous recombination and cell-cycle state [37,38]. Furthermore, because RAD51 formation is cell-cycle dependent, differences in the late RAD51 signal may partly reflect radiation-induced differences in cell-cycle distribution. Confirmation of the observed ordering in other cell types and tissue sources, over a broader dose range, and, if possible, with cell-cycle phase resolution, is a subject of future work.

## 4. Materials and Methods

### 4.1. Cell Culture and Cultivation Conditions

Three primary cultures of human adipose-derived mesenchymal stromal/stem cells (hMSCs) at passages 5–6 were obtained from the Cell Systems LLC collection (database MC16.05.16; accession numbers 250716; 270716; 290716; Moscow, Russia). Cells were cultured in low-glucose DMEM containing 1 g/L glucose (Thermo Fisher Scientific, Waltham, MA, USA), supplemented with 10% fetal bovine serum (Thermo Fisher Scientific, Waltham, MA, USA), penicillin (50 U/mL), and streptomycin (50 μg/mL) (PanEco, Moscow, Russia). Cells were maintained at 37 °C in a humidified atmosphere containing 5% CO_2_, with medium replacement every three days.

### 4.2. Irradiation Conditions and Dosimetry

Cells in the exponential growth phase at approximately 60–70% confluence were irradiated at doses 0.25 Gy, 0.5 Gy, 1.0 Gy, 2.0 Gy at the A. Tsyb Medical Radiological Research Center, a branch of the National Medical Research Radiological Center of the Ministry of Health of the Russian Federation. Three radiation modalities were used: 14.1 MeV neutrons generated by an NG-14 neutron generator (VNIIA, Moscow, Russia; dose rate, 46 mGy/min), γ-rays from a ^60^Co ROKUS-AM gamma-therapy unit (Ravenstvo, Saint Petersburg, Russia; dose rate, 0.5 Gy/min), and 150 MeV protons delivered in the pass-through mode at the Prometheus proton therapy complex (Lebedev Physical Institute, Moscow, Russia; time-averaged dose rate 1 Gy/min). The proton beam was pulsed and delivered using a scanning irradiation mode.

The track-averaged linear energy transfer (LET) values were approximately 0.2 keV/µm for ^60^Co γ-rays, 0.5 keV/µm for 150 MeV protons, and 12 keV/µm for 14.1 MeV neutrons.

Neutron-field dosimetry was performed using an RBN fast-neutron radiometer (ITER-Center, Moscow, Russia). For proton irradiation, dose-field uniformity and field size were controlled using a PTW Octavius 1600XDR matrix detector, Freiburg im Breisgau, Germany, and absorbed doses were set using a PTW Tandem XDR electrometer connected to a PTW Advanced Markus TM 34045 (Freiburg im Breisgau, Germany) plane-parallel ionization chamber. Gamma-ray dosimetry was performed according to the IAEA TRS-398 Rev. 1 protocol [43] using a PTW Unidos Webline electrometer and a PTW Farmer TM30013 ionization chamber (PTW, Freiburg im Breisgau, Germany). The reported dosimetric uncertainties were standard uncertainties evaluated according to the methodology described in IAEA TRS-398 Rev. 1 [43]; they did not exceed 5% for neutron and proton irradiation and were 2% for γ-ray irradiation.

After irradiation, cells were incubated under standard conditions at 37 °C and 5% CO_2_. For dose-response measurements, irradiated samples and the corresponding sham controls were fixed at 0.5 h post-irradiation. For kinetic measurements, irradiated and sham-control samples were fixed at 0.5, 2, 4, 6, and 24 h post-irradiation.

Each independent experimental run included a time-matched non-irradiated sham control. Sham-control samples underwent the same handling and transportation procedures as the irradiated samples but were not exposed to radiation. All three radiation modalities were investigated on the same day within each experimental run; therefore, a single common sham-control series was used for the γ-ray-, proton-, and neutron-irradiated groups. Sham-control samples were fixed at the same time points as the irradiated samples (0.5, 2, 4, 6, and 24 h). The complete experiment was conducted independently three times on separate weeks.

### 4.3. Immunocytochemical Staining and Foci Quantification

Cells were fixed with 4% paraformaldehyde in phosphate-buffered saline (PBS; pH 7.4) for 20 min at room temperature. After fixation, samples were washed twice with PBS and incubated for 1 h in 0.3% Triton X-100 in PBS supplemented with 5% goat serum for permeabilization and blocking of nonspecific antibody binding.

Samples were then incubated overnight at 4 °C with primary antibodies diluted in PBS containing 1% bovine serum albumin (BSA). The following primary antibodies were used: rabbit monoclonal anti-γH2AX antibody recognizing phosphorylated Ser139 (clone EP854(2)Y, Abcam, Waltham, MA, USA; dilution 1:800), mouse monoclonal anti-pATM antibody recognizing phosphorylated Ser1981 (clone 10H11.E12, Abcam, Waltham, MA, USA; dilution 1:400), and rabbit polyclonal anti-RAD51 antibody (ABE257, Merck Millipore, Burlington, VT, USA; dilution 1:200).

After incubation with the primary antibodies, samples were washed three times with PBS and incubated for 1 h with secondary antibodies diluted in PBS containing 1% BSA. Goat anti-mouse IgG (H + L) conjugated to Alexa Fluor 488 (Abcam, Waltham, MA, USA; dilution 1:2000) and goat anti-rabbit IgG (H + L) conjugated to Alexa Fluor 555 (Abcam, Waltham, MA, USA; dilution 1:2000) were used as secondary antibodies for pATM and γH2AX staining, accordingly. Goat anti-rabbit IgG (H + L) conjugated to Alexa Fluor 488 (Abcam, USA; 1:2000) were used as secondary antibodies for RAD51 staining. Samples were mounted using ProLong Gold antifade mounting medium containing DAPI (Life Technologies, Carlsbad, CA, USA;1 μg/mL) for nuclear counterstaining and protection against photobleaching.

Fluorescence images were acquired using a Nikon Eclipse Ni-U fluorescence microscope (Nikon, Tokyo, Japan) equipped with UV-2E/C, B-2E/C, and Y-2E/C filter sets. Images were documented using a ProgRes MFcool high-resolution fluorescence microscopy camera (Jenoptik AG, Jena, Germany). Nuclear γH2AX, pATM, and RAD51 foci were quantified using the DARFI software (http://github.com/varnivey/darfi; accessed on 19 September 2016) package and manual scoring. For each marker, experimental condition, and time point, 50 cells were analyzed on each of three slides in every independent experiment. Thus, 150 cells were evaluated per condition and time point in each experiment, corresponding to 450 cells across the three independent experiments.

### 4.4. Cytotoxicity Assessment

Cytotoxicity was additionally evaluated in the same hMSC cultures under irradiation conditions comparable to those used in the main experiments. Cells were exposed to 0.5, 1.0, and 2.0 Gy using the same irradiation facilities: ^60^Co γ-rays from the ROKUS-AM unit, 150 MeV protons from the Prometheus proton therapy complex, and 14.1 MeV neutrons from the NG-14 neutron generator. Irradiated and sham control samples were fixed at 24 h post-irradiation. Cytotoxicity was assessed using the Image-iT™ DEAD Green™ Viability Stain (catalog no. I10291, Invitrogen, Carlsbad, CA, USA), which stains the nuclei of cells with compromised plasma membrane integrity green. For each experimental condition, 100 cells were evaluated by fluorescence microscopy using a 10× objective on each of three slides in every independent experiment, and the percentage of DEAD Green-positive cells was determined. Thus, 300 cells were evaluated per condition and time point in each experiment, corresponding to 900 cells across the three independent experiments.

### 4.5. Experimental Data Preprocessing

The main kinetic analysis was performed for γH2AX and pATM foci measured at 0.5, 2, 4, 6, and 24 h after irradiation at doses of 0.25, 0.5, 1.0, and 2.0 Gy. For descriptive exponential fitting, background-subtracted values were used, calculated by subtracting the corresponding time-matched mean of the common non-irradiated sham control from each irradiated value.

RAD51 kinetics were not used for parameter estimation in the γH2AX/pATM kinetic models. Instead, RAD51 was analyzed separately as a complementary biological readout of late repair-associated activity.

Foci counts were summarized separately for each of the three independent experiments. The reported values are the mean ± standard error (SE) calculated from the three independent experimental means (n = 3). Individual cells and slides were not treated as independent biological replicates. The same common sham-control series was displayed with each radiation modality because γ-ray, proton, and neutron irradiation were performed within the same experimental run. Mean foci counts from the irradiated samples were used for mechanistic kinetic-model fitting, with the corresponding SE values used as experimental uncertainties in the weighted χ^2^ objective function. The first available post-irradiation measurement, obtained at 0.5 h, was used as the initial time point for kinetic modeling. In the kinetic models, irradiation was approximated as an instantaneous input, and the finite duration of dose delivery was not explicitly represented. This simplification should be considered when interpreting the early-time kinetic parameters.

### 4.6. Biexponential Approximation

As an initial descriptive analysis, background-subtracted mean γH2AX and pATM foci counts were fitted separately for each radiation quality and dose using the biexponential function in Equation (1). The amplitudes *A_fast_*(*d*) and *A_slow_*(*d*) were estimated separately for each dose, whereas *τ_fast_* and *τ_slow_* were assumed to be common for a given marker and radiation type.

This approximation was used solely as a descriptive benchmark to assess the presence of multiple kinetic timescales.

### 4.7. Two-Component Baseline Kinetic Model

A two-component baseline kinetic model was constructed using a rapidly repairable component *D_s_* and an effective complex component *D_c_*:

(2) $$\frac{{\mathrm{dD}}_{s}}{\mathrm{dt}}=-k_{\mathrm{rs}}D_{s},$$

(3) $$\frac{{\mathrm{dD}}_{c}}{\mathrm{dt}}=-\frac{v_{\mathrm{rc}}D_{c}}{\left(K_{\mathrm{rc}}+D_{c}\right)}$$

The initial total damage level *N*_0_(*d*) was proportional to absorbed dose. The initial partitioning between the two components was defined as

(4) *D_s_*(0) = *αN*_0_(*d*),

(5) *D_c_*(0) = (1 − *α*)*N*_0_(*d*),

where α denotes the initial fraction assigned to the rapidly repairable component and was estimated separately for each radiation type and dose.

The damage-associated input to ATM activation was defined as a weighted sum of the two components,

(6) Φ(*t*) = *D_s_*(*t*) + *w_dc_D_c_*(*t*),

where *w_dc_* is the effective contribution of the complex component to the DDR signal. ATM activation and γH2AX signal formation were represented by the following effective signaling subsystem:

(7) $$\frac{dA_{f}}{\mathrm{dt}}=\;\frac{V_{\mathrm{af}}\Phi (t)}{K_{m}+\Phi (t)}-\;k_{\mathrm{df}}A_{f},$$

(8) $$\frac{dA_{s}}{\mathrm{dt}}=k_{\mathrm{as}}A_{f}-k_{\mathrm{ds}}A_{s},$$

(9) $$\frac{\mathrm{dH}}{\mathrm{dt}}=k_{p}\left(A_{f}+A_{s}\right)-k_{g}H.$$

Here, *A_f_* and *A_s_* denote the fast and slow components of the pATM signal, respectively, whereas *H* denotes the γH2AX signal. The modeled observables were defined as pATM = *A_f_* + *A_s_* and *γ*H2AX = *H*. The initial signaling variables were set to *A_f_*(0) = *A_s_*(0) = *H*(0) = 0.

The two-component baseline model was fitted jointly to the γH2AX and pATM time courses for γ-rays and neutrons, separately at each dose. Proton data were analyzed using the extended model described below.

### 4.8. Extended Kinetic Model with an Effective Ultraslow Component

The baseline model was extended by introducing an effective ultraslow component *D_u_*. In the extended model, the dynamics of the complex and ultraslow components were described as

(10) $$\frac{{\mathrm{dD}}_{c}}{\mathrm{dt}}=-\frac{V_{\mathrm{rc}}D_{c}}{K_{\mathrm{rc}}+D_{c}}-k_{\mathrm{cu}}D_{c},$$

(11) $$\frac{dD_{u}}{\mathrm{dt}}=k_{\mathrm{cu}}D_{c}-k_{u}D_{u},$$

where *k_cu_* denotes the transition rate from *D_c_* to *D_u_*, whereas *k_u_* denotes the removal rate of the ultraslow component. The damage-associated input to ATM activation was extended accordingly:

(12) Φ(*t*) = *D_s_*(*t*) + *w_dc_D_c_*(*t*) + *s_du_w_dc_D_u_*(*t*).

where *s_du_* is the relative signaling weight of the ultraslow component.

The initial conditions were specified as

(13) *D_s_*(0) = *αN*_0_(*d*), *D_c_*(0) = (1 − *α*)(1 − *π*)*N*_0_(*d*), *D_u_*(0) = (1 − *α*)*πN*_0_(*d*)

Here, *α* denotes the initial fast-component fraction, whereas *π* denotes the radiation-type-specific fraction of the non-fast component assigned to *D_u_*. The normalized initial ultraslow component, where *D_u_*_0_ ≡ *D_u_*(0), was calculated as

(14) $$\frac{D_{u0}}{N_{0}}=\left(1-\alpha \right)\pi .$$

This dimensionless quantity was used as the model-derived late-persistent DDR index.

The extended model was fitted separately at each dose for all three radiation types. At each dose, γ-ray and neutron data were fitted jointly, whereas proton data were fitted separately using the same model structure and optimization procedure. Pooled fits across doses were additionally performed for the γ-ray and neutron datasets to assess the robustness and identifiability of the radiation-quality separation.

### 4.9. Parameter Estimation and Model Comparison

Model parameters were estimated using weighted nonlinear least squares. For each experimental data point *i*, the standardized residual was defined as

(15) $$r_{i}=\frac{y_{i}^{\mathrm{mod}}-y_{i}^{\exp}}{{\mathrm{SE}}_{i}},$$

where $y_{i}^{\exp}$ is the experimental mean foci counts, $y_{i}^{\mathrm{mod}}$ is the corresponding model value, *SE_i_* is the standard error of the mean calculated from the means of the three independent experiments.

The objective function was defined as the sum of squared normalized residuals:

(16) $$\chi^{2}=\sum_{i}r_{i}^{2}.$$

The systems of ordinary differential equations were integrated using the LSODA algorithm implemented in scipy.integrate.solve_ivp, with relative and absolute tolerances of 10^−6^ and 10^−8^, respectively. Parameter optimization was performed using the trust-region reflective algorithm implemented in scipy.optimize.least_squares, subject to parameter bounds. To reduce sensitivity to the initial parameter values, optimization was repeated from multiple starting points, and the solution with the lowest *χ*^2^ value was retained. Model fits were compared using *χ*^2^, the number of fitted parameters, Akaike information criterion (AIC), and the small-sample corrected Akaike information criterion (AICc):

(17) *AIC* = *χ*^2^ + 2k,

(18) $$\mathrm{AICc}=\mathrm{AIC}+\frac{2k(k+1)}{n-k-1},$$

where *k* is the number of fitted parameters and *n* is the number of fitted data points.

### 4.10. Bootstrap and Identifiability Analysis

Parameter uncertainty was assessed separately for each dose using a parametric bootstrap procedure. For each modeled radiation type, marker, and time point, synthetic mean foci counts were sampled from a normal distribution centered on the corresponding experimental mean, with the experimental SE used as the standard deviation. Negative simulated values were set to zero. A total of 100 synthetic datasets were generated for each dose, and each dataset was refitted using the same optimization procedure as the original data. Bootstrap distributions were summarized using the median and the 2.5th and 97.5th percentiles. The robustness of the radiation-quality difference was assessed as the proportion of bootstrap replicates in which the normalized initial ultraslow component was larger after neutron irradiation than after γ-irradiation.

To evaluate parameter interdependence and practical identifiability, pairwise Pearson correlation matrices were calculated from the bootstrap parameter estimates.

### 4.11. Separate Analysis of RAD51 as a Complementary Biological Readout

RAD51 data were analyzed separately and were not used for parameter estimation in the γH2AX/pATM kinetic models. For each radiation type and dose, the residual RAD51 signal was calculated as the ratio of the mean number of RAD51 foci per cell at 24 h post-irradiation to the corresponding time-matched mean of the sham control. The analysis included doses of 0.5, 1.0, and 2.0 Gy. The 0.25 Gy dose was not included because RAD51 measurements were not performed at this dose in the original experimental series.

The relative contributions of radiation type and absorbed dose were assessed using a two-way additive decomposition of the total sum of squares across the radiation-type × dose matrix. Because only one aggregated value was available for each combination of radiation type and dose, the interaction term could not be estimated separately and was included in the residual variation. The contribution of each factor was calculated as its sum of squares divided by the total sum of squares and expressed as a percentage. These percentages were used as descriptive measures rather than as inferential tests.

## 5. Conclusions

In the present study, late γH2AX and pATM persistence in human mesenchymal stromal/stem cells was associated more with radiation quality than with absorbed dose within the investigated range of 0.25–2.0 Gy. Using kinetic modeling of the response to γ-rays, 150 MeV protons, and 14.1 MeV neutrons, we introduced an effective ultraslow component as a model-based index of late-persistent DDR signaling. The normalized initial ultraslow component followed the order γ-rays < protons < neutrons across all investigated doses.

Although the absolute values of the ultraslow component in the pooled scheme were only partially identifiable, its ordering by radiation quality was preserved in the per-dose fits, and the γ-ray–neutron separation was supported by parametric bootstrapping. RAD51, analyzed separately and not included in model fitting, showed the same qualitative ordering at 24 h and therefore provided complementary biological support for the radiation-quality dependence of late DDR persistence. The concordance among the measured γH2AX and pATM kinetics, the model-derived ultraslow index, the late RAD51 response, and track-structure considerations strengthens the conclusion that late DDR-marker persistence varied systematically with radiation quality under the investigated conditions, without establishing a one-to-one correspondence between the fitted component and a specific lesion class.

Future studies should test the robustness of this ordering across additional cell types and tissue sources, a broader dose range, and a denser time course extending beyond 24 h. Combining DDR-marker kinetics with cell-cycle-resolved measurements, long-term viability, clonogenic survival, and chromosomal-aberration assays, together with track-structure modeling and analysis of spatial damage organization, will be necessary to relate the model-derived late component to specific molecular lesions and functional outcomes. Validation in tissue-based or in vivo systems will also be required before the findings can be translated to radiation-risk assessment or clinical radiotherapy.

## Figures and Tables

**Table 1:** Parameters of the two-exponential approximation for γH2AX and pATM foci kinetics after subtraction of the control level.

Marker	Radiation Type	*τ_fast_*, h	*τ_slow_*, h	χ^2^
γH2AX	γ-radiation	0.32	6.67	2.45
γH2AX	14.1 MeV neutrons	3.85	>200	4.17
pATM	γ-radiation	2.48	11.35	1.94
pATM	14.1 MeV neutrons	1.64	20.84	3.95

**Table 2:** Parameters and fitting quality of the two-component model by dose: χ^2^, α (fast-repairing fraction for γ-rays and neutrons), and *w_dc_* (effective weight of the complex fraction in the ATM activation signal).

Dose, Gy	χ^2^	df	*α_γ_*	*α_n_*	1 − *α_γ_*	1 − *α_n_*	*w_dc_*
0.25	9.206	7	0.662	0.458	0.3383	0.5420	0.9164
0.50	7.743	7	0.947	0.796	0.0533	0.2041	0.0950
1.00	18.560	7	0.021	0.051	0.9787	0.9488	3.7912
2.00	22.592	7	0.621	0.240	0.3794	0.7605	0.2707

**Table 3:** Per-dose estimates of *D_u_*_0_/*N*_0_ for γ-rays, protons, and neutrons at doses 0.25–2.0 Gy, along with the corresponding χ^2^ values of the fit.

Dose, Gy	χ^2^	df	*D_u_*_0_/*N*_0_, *γ*	*D_u_*_0_/*N*_0_, *p*	*D_u_*_0_/*N*_0_, *n*
0.25	12.69	8	0.087	0.178	0.507
0.5	9.19	8	0.083	0.094	0.233
1.0	17.18	8	0.066	0.101	0.264
2.0	24.71	8	0.014	0.033	0.214

**Figure A1:**
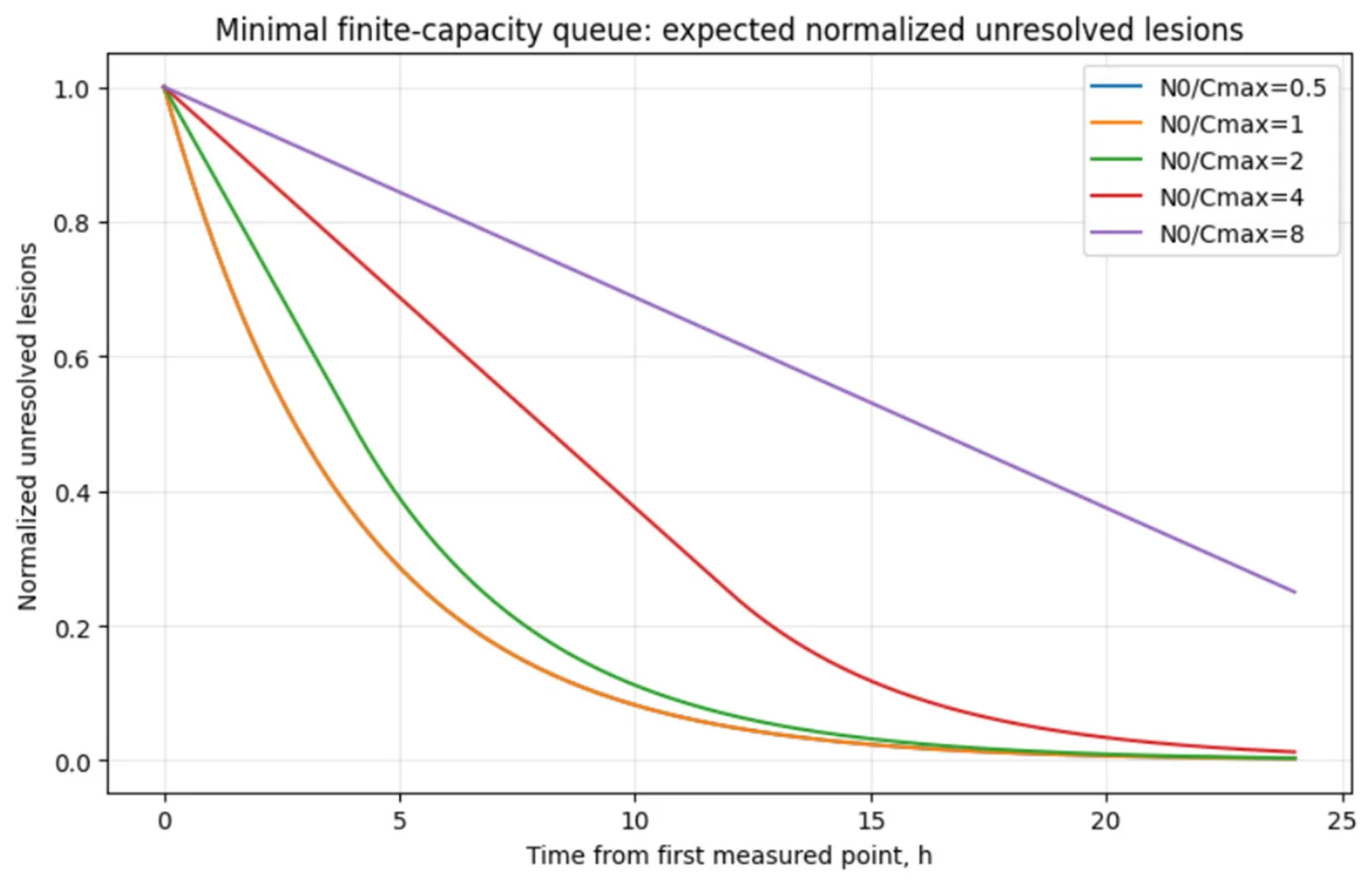
Minimal finite-capacity queue model: expected normalized number of unresolved lesions for different degrees of overload *N*_0_/*C*_max_.

**Figure A2:**
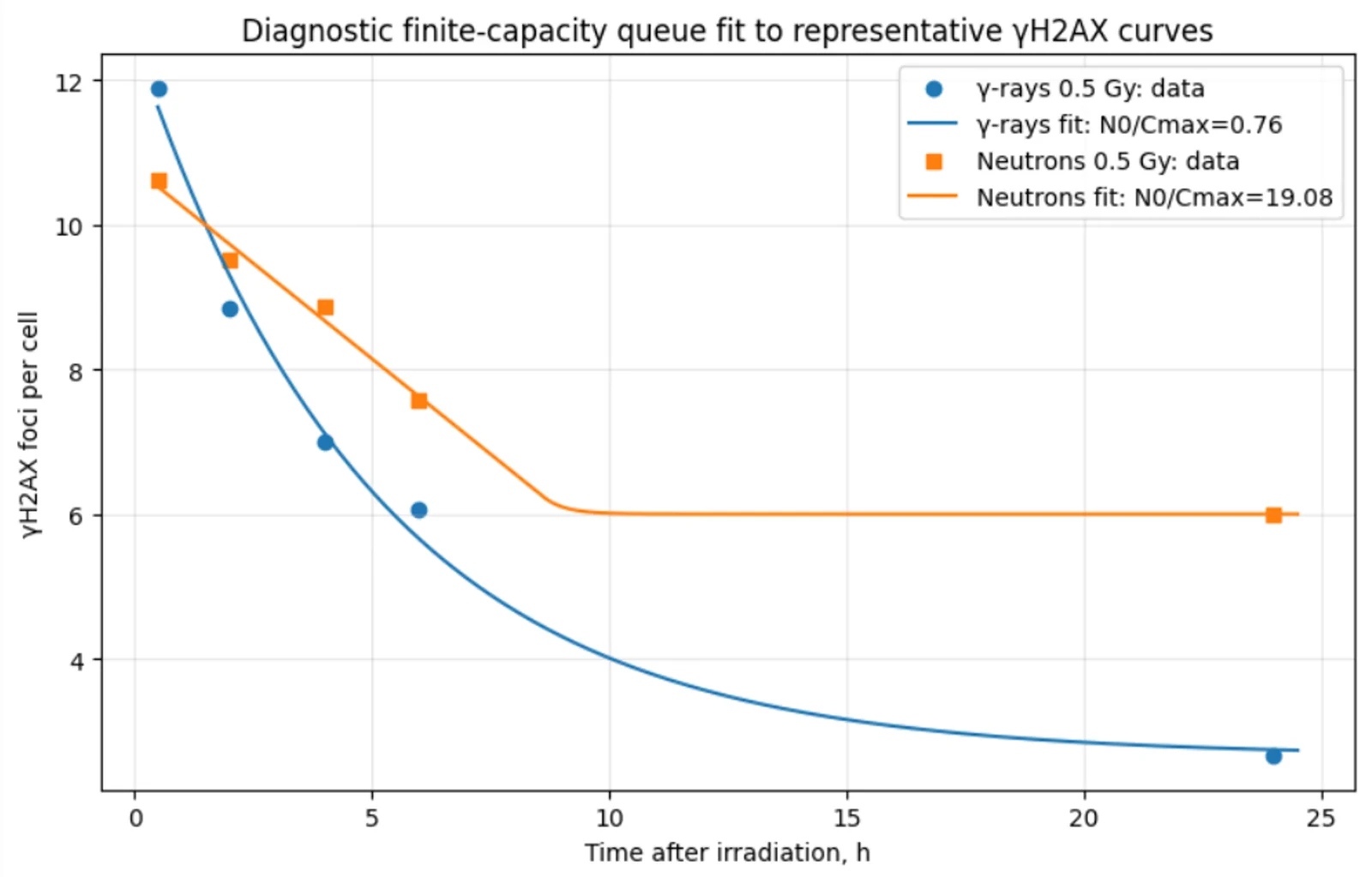
Diagnostic fit of the queue model to representative γH2AX curves at 0.5 Gy for γ-rays and neutrons.

**Figure A3:**
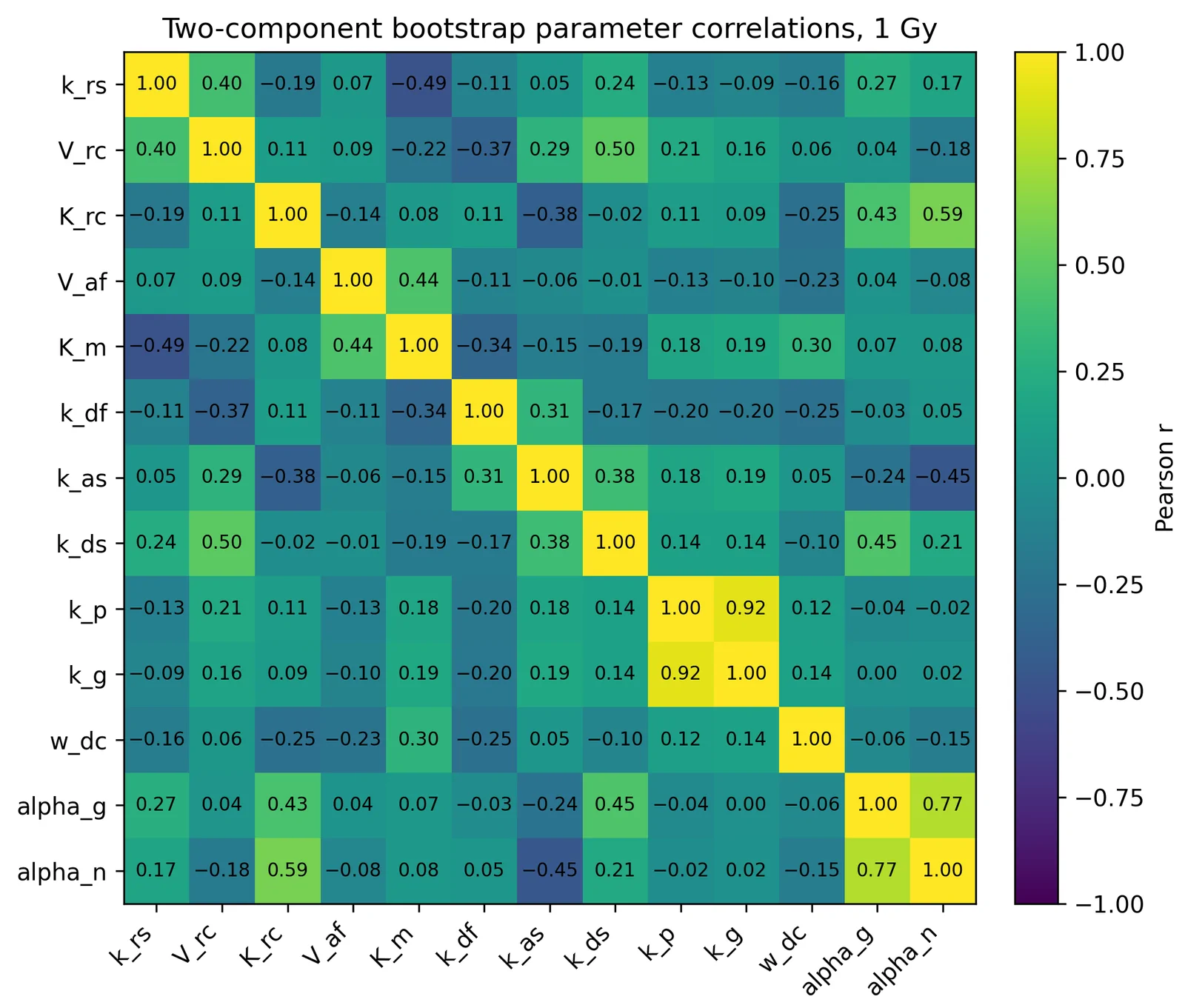
Correlation matrix of parameters of the two-component model (bootstrap), dose 1.0 Gy.

**Figure A4:**
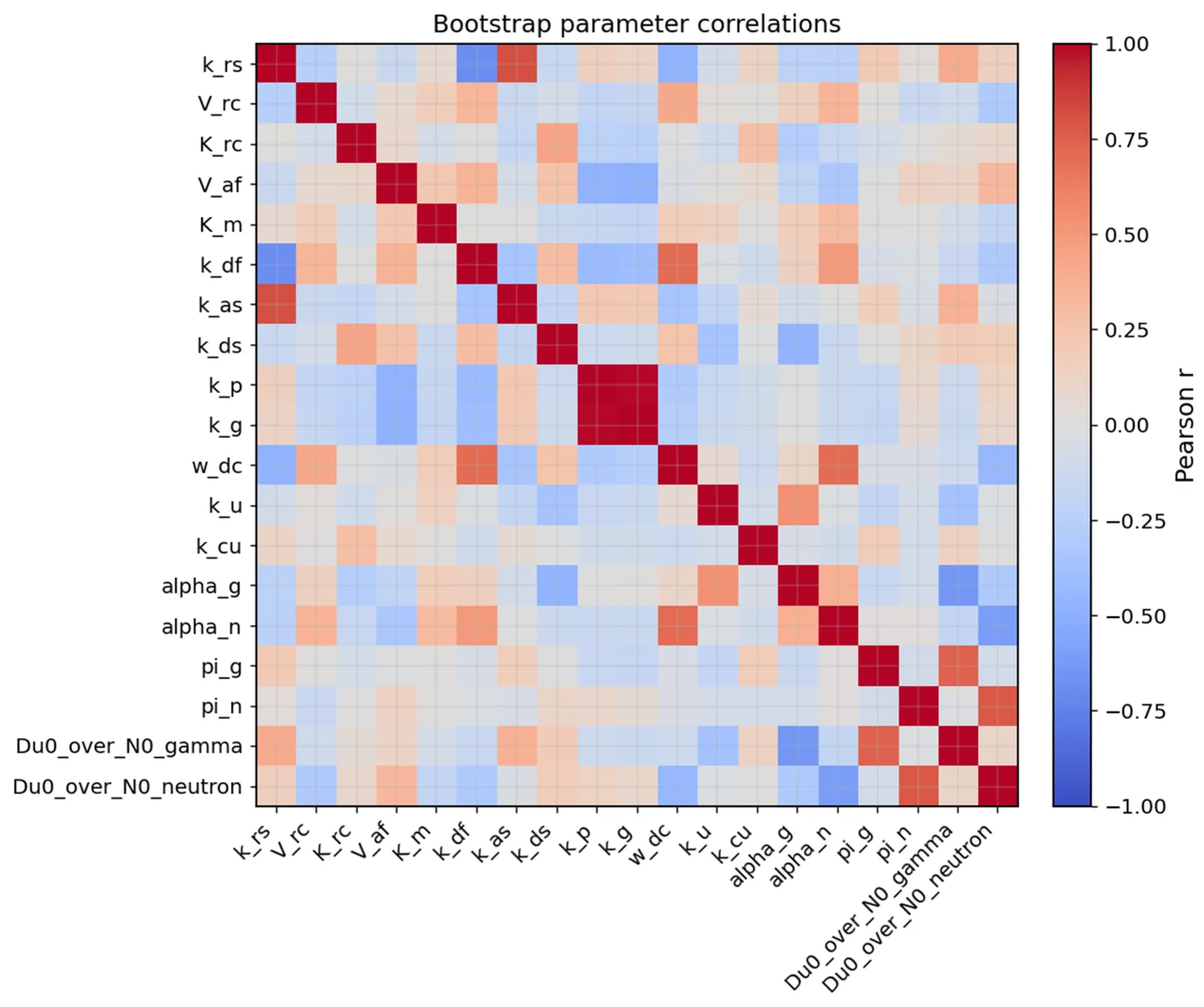
Correlation matrix of parameters of the three-component model (bootstrap, joint fitting).

## Data Availability

The original contributions presented in this study are included in the article. Further inquiries can be directed to the corresponding author.

## Abbreviations

The following abbreviations are used in this manuscript:

AIC	Akaike information criterion
AICc	Akaike information criterion corrected for finite sample size
ATM	Ataxia-telangiectasia mutated kinase
df	Degrees of freedom
DDR	DNA damage response
DSB	DNA double-strand break
Gy	Gray
hMSC	Human mesenchymal stromal/stem cell
LET	Linear energy transfer
NHEJ	Non-homologous end joining
ODE	Ordinary differential equation
pATM	Phosphorylated ATM
RAD51	RAD51 recombinase
RIF	Radiation-induced focus
SEM	Standard error of the mean
γH2AX	Phosphorylated histone H2AX at Ser139

## Appendix A. Diagnostic Test of the Minimal Finite-Capacity Markov Queue Model

### Appendix A.1. Motivation

In the discussion of late persistence of γH2AX and pATM signals, an alternative interpretation arises according to which prolonged signal retention may be attributable not only to lesion complexity but also to limited throughput capacity of the repair system: lesions pass through a finite number of processing units, and under overload, some lesions are forced to wait for processing. To test whether such a mechanism alone can generate a late residual signal without introducing an explicit ultraslow component, we examined a minimal Markov queue model with finite capacity.

### Appendix A.2. Model

Lesions proceed through a sequence of states:

(A1) *W* → *P* → *R*,

where *W* denotes lesions waiting for processing, *P* lesions being actively processed, and *R* resolved lesions. In the fast-loading limit (*k*_on_ ≫ *k*_rep_), where free processing capacity is immediately filled by waiting lesions, the system can be described in terms of the total number of unresolved lesions *U*(*t*) = *W*(*t*) + *P*(*t*). With a finite capacity *C*_max_ limiting the number of lesions processed simultaneously, the dynamics are governed by

(A2) $$\frac{\mathrm{dU}}{\mathrm{dt}}=-k_{\mathrm{rep}}\min\left(U,C_{\max}\right).$$

When the number of lesions is below capacity, all are repaired in parallel and the decay is exponential; when capacity is exceeded, the system is saturated and an approximately linear “queue clearance” occurs first. The key dimensionless parameter is the ratio of the initial load to the throughput capacity, *N*_0_/*C*_max_, which characterises the degree of overload.

For comparison with experiment, we used the observation model

(A3) *S*(*t*) = *S*_∞_ + *A* *u*(*t*),

(A4) *u*(*t*) = *U*(*t*)/*N*_0_,

where *S*_∞_ is the residual signal level and *A* is the amplitude of the queue-dependent signal component. The fitted parameters were *N*_0_/*C*_max_, *k*_rep_, *S*_∞_, and *A*.

### Appendix A.3. Model Behavior

Figure A1 shows the normalized number of unresolved lesions U(t)/*N*_0_ for values of *N*_0_/*C_max_* = 0.5,1,2,4,8 with a fixed *k*_rep_ = 0.25 h^−1^. Under moderate load, the decay is close to exponential; under severe overload (*N*_0_/*C*_max_ = 8), the decay is markedly slowed, and about one quarter of the initial signal remains at 24 h.

### Appendix A.4. Fitting to Representative γH2AX Curves

The model was applied as a diagnostic fit to two representative γH2AX curves at 0.5 Gy (γ-rays and neutrons), using time points 0.5, 2, 4, 6, and 24 h; time in the model corresponds to 0.5 h post-irradiation. The fitted parameters are summarized in Table A1, and the corresponding curves are shown in Figure A2.

**Table A1:** Fitting results.

Parameter	γ-Radiation	Neutrons
*N*_0_/*C*_max_(overload)	0.76	19.08
*k_rep_*, h^−1^	0.136	0.864
*S*_∞_, foci/cell	1.8	≈6.0
A, foci/cell	10.18	4.53
SSE	0.46	0.10
Observed 24 h signal, foci/cell	2.65	6.00
Predicted 24 h signal, foci/cell	2.74	6.00

### Appendix A.5. Interpretation

For γ-rays, the model behaves naturally: *N*_0_/*C*_max_ ≈ 0.76, meaning that the processing capacity is not overloaded, and the curve corresponds to a regular decay with a small residual level. For neutrons, the model is forced to assume *N*_0_/*C*_max_ ≈ 19—the initial load is nearly 19 times the simultaneous throughput capacity. Moreover, the late residual neutron signal does not arise naturally from the queue dynamics but is effectively imposed by a large residual level *S*_∞_ ≈ 6.0.

Thus, to approach the neutron curve, the minimal queue model required simultaneously a biologically implausible overload and an externally imposed late level. This shows that the limited-capacity model in the tested form does not generate an autonomous late persistent component: finite capacity may be one contributing factor, but describing the late residual signal requires an additional source of slow kinetics—lesion heterogeneity, complex/clustered breaks, chemically modified ends, chromatin-dependent accessibility, or prolonged end processing. This motivated the introduction of the ultraslow component as an explicit effective element of the model.

## Appendix B. Parameter Correlations and Identifiability

### Appendix B.1. Purpose

To assess the identifiability of the parameters of the kinetic models, pairwise Pearson correlation coefficients between fitted parameters were computed from bootstrap replicates. Strong correlations indicate that the parameters are not determined independently by the data, but only in combination, reflecting partial non-identifiability.

### Appendix B.2. Two-Component Model

The correlation matrix for the parameters of the two-component model (dose 1.0 Gy, as a representative example) is shown in Figure A3. Pronounced correlations are observed between several kinetic and signaling parameters: different combinations of them yield similar goodness-of-fit. This is consistent with the multimodality and partial non-identifiability of absolute parameter values noted in Section 2.5.

### Appendix B.3. Three-Component Model

The correlation matrix for the parameters of the main three-component model (joint fitting) is shown in Figure A4. As in the two-component model, individual parameters are correlated; in particular, the fraction of the ultraslow component and the fraction of the fast component are related within each radiation type, which is expected because the normalized ultraslow fraction is determined by their combination, namely *D_u_*_0_/*N*_0_ = (1 − *α*)*π*.

Crucially, however, the estimates of the target quantity *D_u_*_0_/*N*_0_ for γ-rays and neutrons are practically uncorrelated with each other (*r* ≈ 0.1). This means that the separation of radiation types by the ultraslow fraction is not a consequence of a global parametric correlation, but reflects independent information contained in the data for each type.

Parameter notation used in Figure A3 and Figure A4.

k_rs—repair rate constant of the rapidly repairable damage component *D_s_*.
V_rc—maximal repair rate of the complex damage component *D_c_*.
K_rc—half-saturation constant for saturable repair of *D_c_*.
V_af—maximal rate of fast ATM activation.
K_m—half-saturation constant of ATM activation.
k_df—decay rate constant of the fast pATM component.
k_as—transfer rate constant from the fast to the slow pATM component.
k_ds—decay rate constant of the slow pATM component.
k_p—γH2AX signal formation rate driven by pATM.
k_g—γH2AX signal decay rate.
w_dc—effective signaling weight of the complex damage component *D_c_*.
k_u—repair rate constant of the ultraslow component *D_u_*.
k_cu—transition rate from *D_c_* to *D_u_*.
alpha_g and alpha_n—initial fast-component fractions for γ-rays and neutrons, respectively.
pi_g and pi_n—fractions of the non-fast damage pool assigned to the ultraslow component for γ-rays and neutrons, respectively.
Du0_over_N0_gamma and Du0_over_N0_neutron—normalized initial ultraslow components for γ-rays and neutrons, respectively, calculated as (1 − *α*)*π*.

### Appendix B.4. Conclusions

In both models, the absolute values of individual parameters are only partially identifiable, which is a general property of effective kinetic models of this class. At the same time, in the three-component model, the separation of γ-rays and neutrons by the ultraslow fraction was not driven by a single global parametric correlation, because the corresponding estimates were practically uncorrelated. This supports the approach adopted in this study: conclusions are based on the robust radiation-quality ordering rather than on the literal values of individual parameters.
